# Taxonomic analysis of metagenomic data with kASA

**DOI:** 10.1093/nar/gkab200

**Published:** 2021-03-30

**Authors:** Silvio Weging, Andreas Gogol-Döring, Ivo Grosse

**Affiliations:** Institute of Computer Science, Martin-Luther University Halle-Wittenberg, Von-Seckendorff-Platz 1, Halle, Germany; Department of Mathematics, Natural Sciences and Computer Science, TH Mittelhessen University of Applied Sciences, Wiesenstraße 14, Gießen, Germany; Institute of Computer Science, Martin-Luther University Halle-Wittenberg, Von-Seckendorff-Platz 1, Halle, Germany

## Abstract

The taxonomic analysis of sequencing data has become important in many areas of life sciences. However, currently available tools for that purpose either consume large amounts of RAM or yield insufficient quality and robustness. Here, we present kASA, a *k*-mer based tool capable of identifying and profiling metagenomic DNA or protein sequences with high computational efficiency and a user-definable memory footprint. We ensure both high sensitivity and precision by using an amino acid-like encoding of *k*-mers together with a range of multiple *k*’s. Custom algorithms and data structures optimized for external memory storage enable a full-scale taxonomic analysis without compromise on laptop, desktop, and HPCC.

## INTRODUCTION

Decoding the complex composition of microbial communities is important, for example, to determine Earth’s biodiversity or its role in human diseases. However, this poses a major challenge. One of the biggest problems is the sheer number of different organisms living together in communities. Most organisms thrive only in the community of other organisms so specific cultivation of a single species is often challenging (1). Microbial communities therefore must be analyzed as a whole which is done by sampling and sequencing the extracted DNA forming the metagenome (2). The technology of next-generation sequencing (NGS) offers the possibility of creating vast amounts of DNA sequences (so called ‘reads’) from the metagenome, usually several million per dataset, each with a length between tens and thousands of nucleotides (3). The processing and taxonomic identification of these reads requires special bioinformatics methods.

In recent years, several tools that are capable of comparing reads to a database containing genomic sequences of known organisms have been published (4). Apart from the investigation of microbial communities, most of these tools can also be used to detect and quantify contamination in any sequencing data, so they can be extremely valuable for data quality assurance in most application areas of NGS.

One of the best known programs for comparing sequences to large databases is MegaBLAST (5), which uses a seed-and-extend heuristic for finding local alignments between a read and the database. While this tool has a high usability and accuracy, it is not very well suited for quickly processing large numbers of sequences, since the analysis of complete NGS datasets using MegaBLAST would require high computational effort. Other tools like Kraken (6), KrakenUniq (7) and Clark (8) use a more time-efficient approach called *k-mer sampling* that compares short sub-sequences of a fixed length *k* (*k*-mers) taken from the reads with a pre-computed index derived from the database. Kraken uses a lowest-common-ancestor (LCA) approach to infer taxonomic membership, whereas Clark needs a fixed taxonomic rank beforehand. Since these methods rely on a hash table they allow very fast identification but require the index to be in primary memory using large amounts of random-access-memory (RAM), sometimes >100GB. Other solutions try to balance between time, accuracy and memory consumption like Centrifuge (9) (via FM-Index (10) and BWT (11)), Kraken2 (12) (via a compact hash table and minimizers), mash (13) (via MinHash (14)), sourmash (15) (via MinHash (14) and ability to use protein sequences), MetaCache (16) (via MinHash (14)), Ganon (17) (via interleaved Bloom filters (18)), Kaiju (19) (via FM-Index (10) and BWT (11) as well as usage of protein sequences), and many more. However, due to the still growing number of reference genomes in, for example, the NCBIs nucleotide sequence database (20), the amount of primary memory required by almost all of these tools finally scales beyond the scope of, for example, a conventional laptop. This means that the user is forced to rely on special and expensive hardware to perform metagenomic analysis.

In this paper, we introduce kASA (*k*-mer Analysis of Sequences based on Amino acid-like encoding), a robust, accurate and deterministic *k*-mer based tool for the analysis of metagenomic sequencing data with a customizable memory footprint. The low primary memory requirement is achieved by an index that does not need to reside completely in the primary memory (RAM) but can remain largely on secondary memory like hard disk or solid state disk (using data structures from (21)). With this feature, kASA is, to the best of our knowledge, the first tool capable of analyzing large metagenomic datasets with superior accuracy within a reasonable period of time, even with large, comprehensive databases and input files in a memory-restricted environment. kASA is written in C++, free, open source and available for all mainstream operating systems (Linux, Mac, Windows) and platforms (laptop, desktop, HPCC) that support 64-bit computing and offer at least 5GB RAM.

Additionally, unlike most other *k*-mer based tools, kASA constructs *k*-mers using an amino acid-like encoding by converting triplets of nucleotides —regardless of coding or non-coding— via a translation table that by default corresponds to the genetic code. This way, storing *k*-mers requires less space (five bits per nucleotide triplet instead of six), and in addition the sensitivity is improved as synonymous DNA mutations (i.e. those that do not affect the encoded amino acid) no longer affect the matching process in at least one of the six possible reading frames. In at least one of the other frames, a nucleotide-level change typically results in a change in the corresponding amino acid sequence therefore precision is maintained (see supplementary file A for a proof). As long as there are differences at the nucleotide level, kASA can distinguish between, for example, homologous protein sequences in different species.

Another feature of kASA is the use of an entire range of word lengths *k*. Most other *k*-mer based methods use only a single *k* and thus have to make a singular decision how to the balance between sensitivity (small *k*) and precision (large *k*). For each run of kASA, the user can select the range for *k* by specifying the lower and upper bounds of the interval. The maximum value for *k* currently is 25, which corresponds to a length of 75nt in the nucleotide space, the lowest value is one. By dynamically adapting the word length *k*, kASA is able to optimize both sensitivity and precision: If a *k*-mer is too short for being specific for a particular organism it may become specific with a longer word length. On the other hand, if a longer *k*-mer cannot be found in the index due to a mutation, it could possibly still be found when using a shorter word length. The index only consists of *k*-mers of maximum length and thus does not depend in the choice of the interval of *k*’s.

With these techniques, kASA can outperform other tools in many cases regarding robustness and accuracy while keeping the primary memory consumption highly customizable.

## MATERIALS AND METHODS

### Index creation

**Figure 1.**
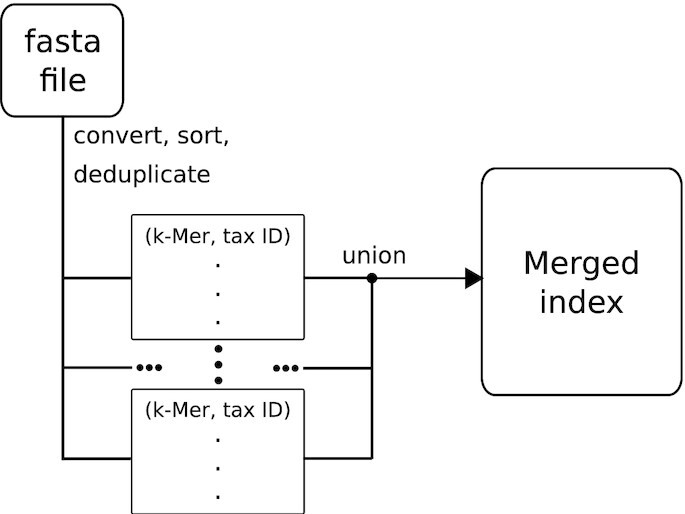
Flowchart for creating the index. In the first step, as much content from one or more fasta files as possible (determined by the memory restricting parameter) is transformed, sorted, and stored into a container of *k*-mers which lies in secondary memory. This step is repeated until no more data is left and at the end, the resulting containers are merged into the final index.

Pre-processing a database to create an index ensures that only necessary calculations are performed while identifying or profiling NGS data. This step (named ‘build’ in kASA) converts a nucleotide or protein database in fasta format to a binary file containing *k*-mers and saves it to secondary memory, for example, the hard drive. The index building step in kASA is depicted in Figure 1. kASA currently supports either 12 or 25 as maximum *k*, which means that 36 or 75 bases make up a *k*-mer (since three bases make up a triplet and this is encoded in one amino acid). These numbers result from the size of an integer (either 64 or 128 bits) in which a *k*-mer is stored. Choosing the latter results in a larger index, but allows a larger range, which is advantageous for long reads. The process is as follows:

In case of DNA, the sequences from the database are scanned in one, three, or six reading frames (depending on user choice, the latter is default) and converted and encoded via a given table to amino acid-like sequences, including coding and non-coding regions. The standard DNA codon table is used by default but this can be changed by the user (hence the amino acid-*like* encoding). In three frames, this translation is lossless except for the first and last base (see supplementary file A).

From the translated sequences all overlapping *k*-mers (i.e. *k* = 12 or *k* = 25 amino acids) are extracted and saved into a file stored in secondary memory, together with the taxonomic ID of their source. To get this ID, we use accession numbers from the fasta files and the NCBI taxonomy database. If there are no accession numbers or taxonomic IDs available, a dummy ID is given and the user receives a notification. Should two or more *k*-mers be identical and have the same taxonomic ID, only one of them is kept and the others are discarded. Note, that this implies having multiple taxa for the same *k*-mer is allowed. The taxonomic level must be specified beforehand much like in CLARK. The lowest level of ‘strain’ or ‘sequence’ is called ‘lowest’ in kASA. The resulting so called ‘content file’ is human readable and can be manually modified if necessary. After sorting and merging redundant entries (those *k*-mers and taxonomic ID), the index is ready to be used for identifying or profiling NGS data. In order to avoid having to rebuild the index every time the database is changed, kASA supports the addition of new reference sequences to the existing index without rebuilding.

Accessing this file during a comparison would create a lot of slow I/O operations, even when using an SSD. To keep the number of these operations minimal and at the same time enable a free choice of the values for *k*, we build a prefix trie (22) from the index containing the first six coded letters of each *k*-mer. Each leaf of this trie contains two integers representing the upper and lower boundaries of the range containing *k*-mers that share the same prefix. If this prefix is matched, only a fraction of the index must be searched for the remaining suffix. This significantly reduces the search space and since the trie is small enough to fit into primary memory, prefix matches are performed very quickly. We therefore set a default lower *k* of seven so that the trie can fully reduce the search space. Finally, we would like to point out that our data structure cannot be a hash table due to our approach of using multiple *k*’s. We are therefore using an stxxl::vector as an underlying data structure.

### Shrink

The index building described in the previous section is designed with no information loss, low access times, and low computational overhead in mind but may result in a large index (up to multiple TB). One reason for this is the introduction of a certain redundancy because two or more identical *k*-mers can have different taxonomic IDs but they still use the space of two or more whole pairs. Unfortunately, this is necessary because the underlying data structure of an stxxl::vector requires uniform space for every entry. While we argue that kASA was not designed to use compact indices because space on secondary memory is more abundant than on primary memory, we decided to give the user the possibility of shrinking the index.

One such possibility is having constrains which enable shrinking the index without information loss. These constrains are a content file with no more than 2^16^ − 1 = 65535 entries, a lower *k* of at least 7, and a 64 bit integer size index which remains static (meaning it is not updated). Since the first six letters of every *k*-mer are already inside the prefix trie, we can delete these letters and instead of saving the taxonomic ID directly to enable updating, we save the line number of the content file. This results in an index half the size but disables updating and binds the content file to this index. This is also done implicitly in case the index is loaded into primary memory: should the constrains be true, only the second half of every 12-mer and the corresponding content line number to its taxonomic ID is saved into the primary memory container. We applied these methods to all viable indices with 64 bit integer size during our benchmarks.

Another method is to randomly delete *k*-mers from the index with a fair amount for each taxon. If a percentage is given, the corresponding number of *k*-mers per taxon is deleted so taxa that consist of more *k*-mers lose more than those that have only a few *k*-mers. Instead of a percentage, a desired size in GB can be specified instead and the index is shrunken to this size by deleting the corresponding calculated percentage. This is of course accompanied by a loss of accuracy and robustness although the effect is not as strong if the sequencing depth is high. This method can also be applied during ‘build’. Supplementary file A contains an experiment that shows the effect of deleting certain percentages from the index with data from the first benchmark described below.

### Identification and profiling

After building the index, the identification of NGS data can be performed. The identification algorithm is as follows:

First, the DNA (and its reverse complement), or already converted amino acid sequences from a fastq or fasta file are (translated and) converted into *k*-mers in a similar way as in ‘build‘. While this is mainly an algorithmic necessity for the following algorithm, this method resembles ‘double indexing’ (23,24). The user may choose between using six, three, or one frame(s). The more frames are used, the longer identification will take (time increases linearly).

**Figure 2.**
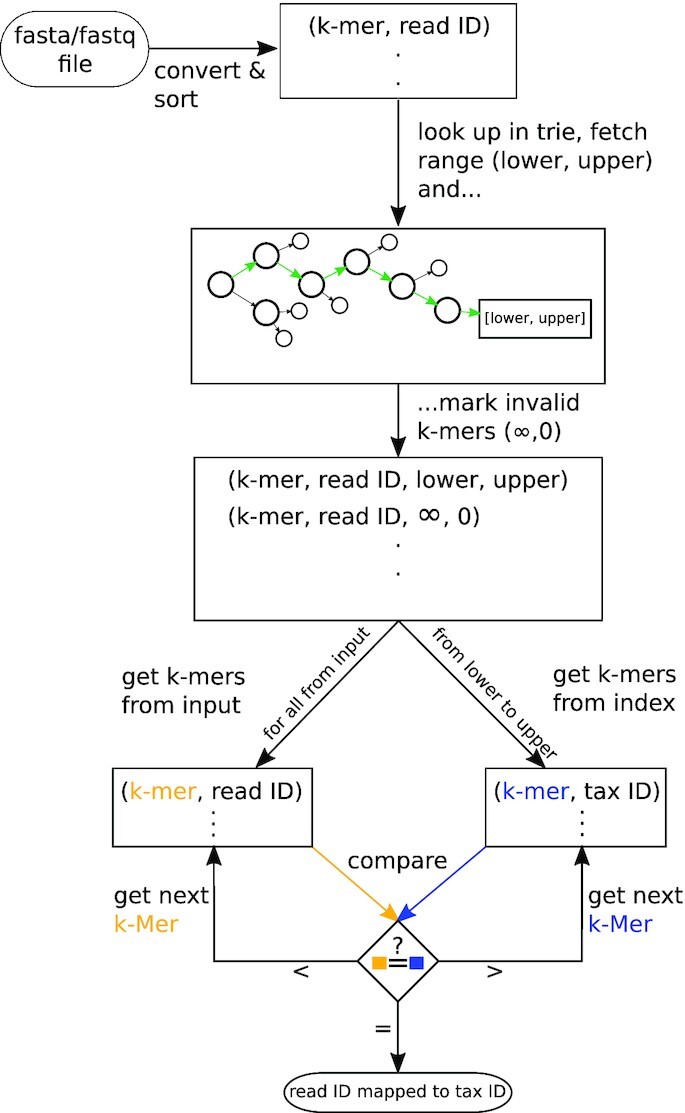
Flowchart of the identification algorithm. The DNA and its reverse complement are converted into pairs of *k*-mer and read ID. After sorting, the prefixes of the *k*-mers determine the suffix ranges in the precalculated index. From this range, the first *k*-mer is compared with the one from the input. If they match, the read ID and the taxonomic ID are scored accordingly. If not, either the next *k*-mer from the input or the next index’ *k*-mer is used. This loop continues until all *k*-mers from the input have been processed.

**Figure 3.**
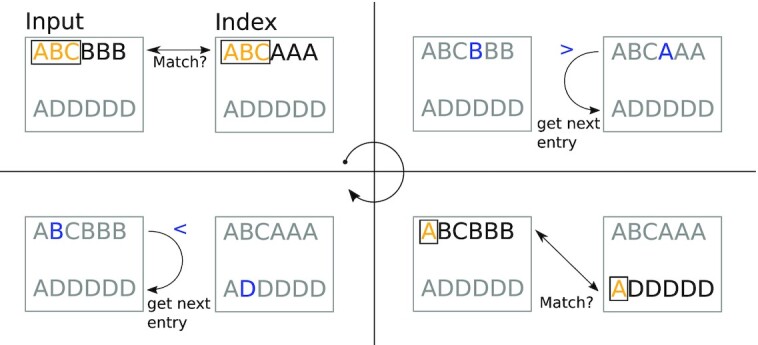
Matching algorithm. First, the *k*-mers are matched for *k* from lowest to highest. In case of a mismatch, either the pointer at the index or the input is iterated further. *k* is set to lowest again and the cycle repeats. A figure with more cases is shown in supplementary file A.

The main difference to the building step is that each *k*-mer receives a read ID instead of a taxonomic ID and that duplicates are kept because they might hint at important motifs. For this reason, we advise the user and reader to de-duplicate reads beforehand to not distort abundances. The pairs are then sorted and passed to a set-intersection-like identification algorithm to see which taxa match to which read, starting with the lower *k*. An overview of this process can be seen in Figures 2 and 3. A pseudocode of the algorithm can be found in supplementary file A.

We have also developed a version of this algorithm that makes more efficient use of the available hardware (e.g. on an HPCC) if the reader wants to process multiple files at the same time. Since reading and writing a file can only be performed by a single thread, there are limits to the acceleration of our parallel algorithm by more processors. We therefore use the given resources to process multiple files in parallel instead of sequentially. This mode is also used for our experiments, since both benchmarks provide more than one input file.

Note that if kASA gets a RAM limit, some input files may be too large to be processed at once, so the files are read and processed in pieces. Since our evaluation scheme is a weighted sum, partitioning the input this way does not change the results. If taxonomic profiling is the main goal of a study, scoring reads can be omitted completely, as this speeds up processing by a factor of ∼1.3.

The scores in the identification algorithm are calculated as follows: Every read which shares at least one *k*-mer with the index will be noted to create a relation from read ID to taxonomic ID. Since matches of smaller lengths are less significant than longer ones, this relation is represented with a weighted sum. Weights *w*_*k*_ are gained by a normalized quadratic function to stress a non-linear increase of significance and to strike a balance between rewarding long matches but not devaluing short matches too much. This means, that the values of *k*^2^ are normalized to (0, 1] for *k* = 1, ..., 25. The sum using those *k*-dependent weights is called: *k*-mer Score of the taxon *t* ∈ *T* and read *r* ∈ *R*

$$\begin{equation*} {k}{\rm -mer\,Score}_{t,r}:=\sum _{x\in I}\sum_{k=k_{{\rm lower}}}^{k_{{\rm upper}}}{w_{k}}.\frac{ \left| {{\rm matches\;with}\;x}_{k}(t,r) \right|}{\left|T \right|} \end{equation*}$$

where *I* denotes the *k*-mers from the Input, $\left| {{\rm matches\;with}\;x}_{k}(t,r) \right|$ is the number of *k*-mers that *t* and *r* share and *T* and *R* are the sets of matched tax or read IDs.

Additionally, a second score called ‘Relative Score’ is calculated before writing the results into a file. It puts the *k*-mer Score in relation to the total number of *k*-mers of a taxon *t* present in the index (also called frequency of *t*) and the length of the read *r*:

$$\begin{equation*}{\rm Relative\, Score}_{t,r} :=\frac{\rm {k-mer\,Score}_{t,r}}{1+\log _{2}\left( {\rm length}(r) \cdot {\rm frequency}(t) \right)}\end{equation*}$$

This formula is inspired by the calculation of the *E*-value in BLAST although in this case, a higher score indicates a better hit. The Relative Score can be used to determine the significance of a matched taxon. From our experience, for a read of length 100 everything with a Relative Score smaller than 0.4 can be seen as insignificant and, for example, sorted out during decontamination. For the output, the resulting scores are sorted in decreasing order by this relative score so that a read can have multiple identified taxa but the leftmost one has the highest value.

The taxonomic profile that kASA computes consists of the names, taxonomic IDs and relative frequencies as well as the number of matched *k*-mers of all taxa found in a dataset. The number and relative frequencies are printed for each *k* and for unique and non-unique matches as well as relative to all *k*-mers in the input. This is necessary since the database used to form the index can be redundant for several reasons: For example an entry is named differently although it is identical to an existing one, or a subspecies is identical to another one on the amino acid level. kASA also offers a value describing the degree of redundancy or ambiguity present in the index which helps deciding which frequency to use. Note however, that for comprehensive databases this value may be overshadowed by large genomes.

Unique relative frequencies are calculated by dividing the number of uniquely matched *k*-mers for a taxon *t* by the total number of uniquely matched *k*-mers:

$$\begin{equation*} h_{U}\left({\rm Taxon}\ t \right)_{k} := \frac{ {\rm unique\; count}(t)}{\left| {\rm uniquely\; matched\; k-mers}\right|} \end{equation*}$$

If a *k*-mer *x* matched multiple taxa, the hit count (how often that *k*-mer matched) is divided by the number of matched taxa. The sum of these floating point numbers is divided by the total amount of matched *k*-mers at the end. The formula is as follows:

$$\begin{equation*} h\left({\rm Taxon} \ t\right)_{k} := {\frac{\sum _{x \in I} \frac{ {\rm hit\; count}(x,t)}{\left|T\right|}}{\left| {\rm matched\; k-mers}\right|}} \end{equation*}$$

If for example *x* was found five times in three different taxa, each of these three would receive $\frac{5}{3}$ to its dividend.

The so called ‘overall relative frequency’ takes the same numerator as the non-unique relative frequency but divides it by the total number of *k*-mers in the input file. This is particularly useful when a file contains a lot of unknown DNA (from the index’ point of view), as it prevents over-interpretation of high-ranking taxa in other relative frequencies.

In conclusion, the identification file offers an answer to the question which organisms were found for each read, creating a basis for further studies. The taxonomic profile provides a broad overview of which organisms are present in the NGS dataset and how much DNA was contributed by them.

## RESULTS

**Figure 4.**
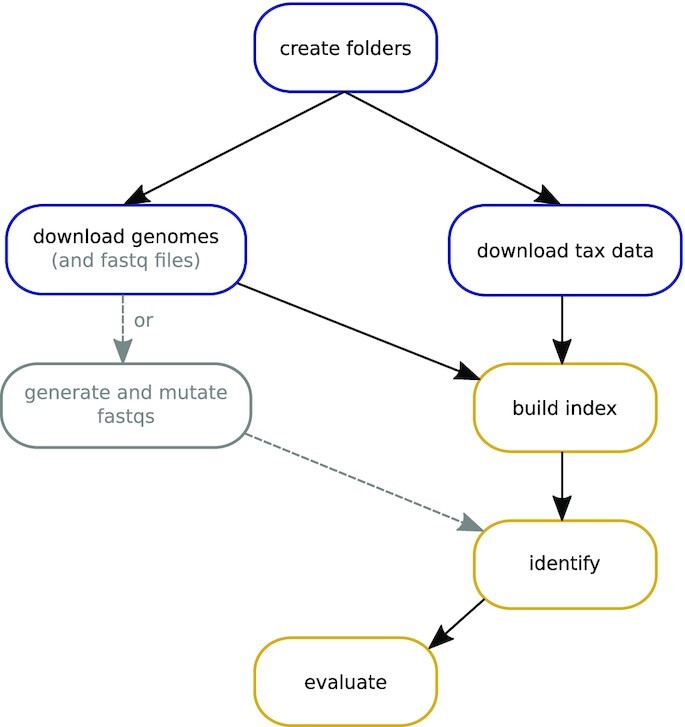
Workflow of both snakemake benchmarks. The steps ‘build’, ‘identify’ and ‘evaluate’ (yellow) are carried out for every tested tool. The other steps (blue) are called only once. The gray box and arrows display a step only used in the robustness benchmark but not in the CAMI benchmark whereas in the box ‘download genomes’ the gray subtext ‘(and fastq files)’ is only used in the latter benchmark.

This section is divided into three parts: First, we test the accuracy and robustness of the identification per read using kASA and a selection of tools mentioned in the introduction with synthetic data. Second, we compare how accurate the created profile is using synthetic data from the CAMI Challenge (25) together with OPAL (26). We created snakemake (27) pipelines for both experiments with synthetic data to enable reproducibility: https://github.com/SilvioWeging/kASA_snakemake and https://github.com/SilvioWeging/kASA_cami, see Figure 4 for a flowchart of the general workflow inside both benchmarks. At the end of this section, we show results from applying kASA to real data and compare the resulting profile to those of Kraken2 and Centrifuge. Additional experiments going beyond the scope of this section can be found in our supplementary file A. Detailed descriptions of our evaluation methods, the tools used and their versions can also be found there. We only chose a subset of the tools mentioned in the introduction for both benchmarks by picking at least one candidate per method. While the pipelines can be expanded to include other organisms and tools, we would like to refer the reader to a more comprehensive benchmark given in (28).

### Taxonomic identification of simulated reads

**Figure 5.**
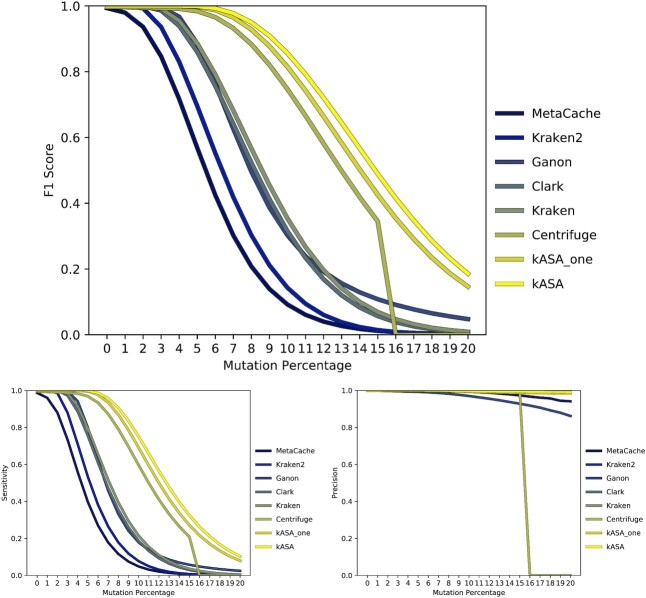
F1 score (top), sensitivity (bottom left) and precision (bottom right) of tested tools for simulated data. Because all tools were used with default settings, kASA is shown with default settings of *k* ∈ [7, 12] and three/one frame(s) as well.

To evaluate the accuracy, performance, and robustness of kASA and mentioned state of the art tools, we designed a synthetic benchmark with snakemake. This benchmark uses the largest chromosomes (or whole genomes in the case of prokaryotes) of multiple model organisms as a reference. From this reference, reads of length 100 are randomly sampled and randomly mutated via a mutation percentage (up to 20%). This simulates the problem of identifying species in data that is noisy, either because of the sequencing technology used or because of real differences to the reference due to mutations or relationship to known species. Results are shown in Figure 5 and can be found in supplementary file B. At the same time, the memory consumption during index building and identification as well as the wall-clock times are logged and shown in Figures 8 and 9.

**Figure 6.**
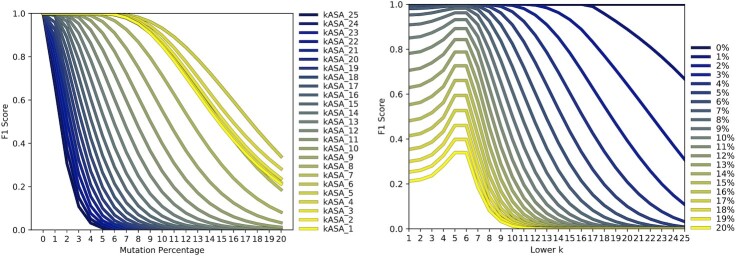
Left: Results for the F1 score with all lower *k*’s from 1 to 25 with a 128 bit index. Right: The same but with lower *k*’s on the x-axis and percentages as graphs.

The plots in Figure 5 show the F1 score, sensitivity, and precision of selected tools for different mutation percentages. Because Kraken and KrakenUniq have the same values, only one of them is included in the plots. As expected, the values decrease non-linearly and tools allowing gaps perform better than hash table based exact matching algorithms. Centrifuge drops to zero at 16% mutations per read because either the minimum required matching seed length of 16nt is not achieved or an internal threshold for the score is not met. Our tool achieves superior sensitivity even without explicitly modeling gaps. Because of the range of *k*’s there is no loss in accuracy if the current part has a mismatch since a smaller *k* still matches the non-mutated part. Note however, that choosing a *k* too low can decrease precision since by-chance hits overshadow legit matches. This effect is visible in Figure 6. It also shows that there is a subset of *k*’s which performs better than all *k*’s before and after them (five and six). We suspect that this subset depends on the data and thus decided against displaying them in Figure 5. On top of our strategy of multiple *k*’s , we abstract to the amino acid-like level which makes at least one frame not likely to be affected by a single mutation due to robustness against synonymous mutations. This can be seen by the difference between the default version and the one with only one frame. The index was created with six frames to make this possible which is the default for ‘build’.

**Figure 7.**
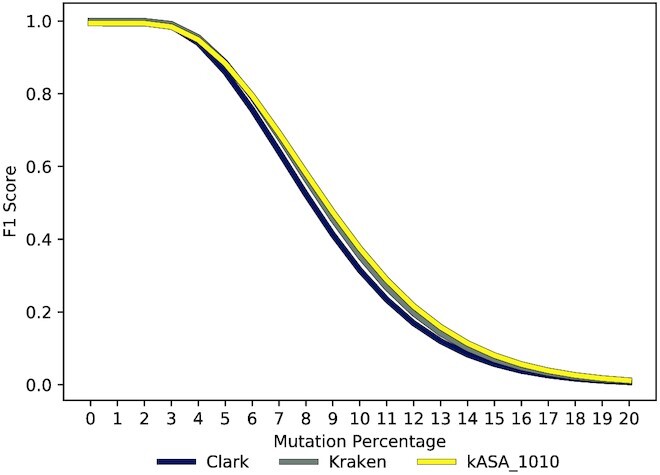
F1 score of CLARK, Kraken, and kASA with a fixed *k* of 10 corresponding to a DNA based tool with *k* = 30.

In its core, kASA is still a *k*-mer based algorithm so we also compared how strong the effect of the amino acid-encoding by itself with a fixed *k* performs against CLARK (*k* = 21) and Kraken (*k* = 31). For this we fixed the upper and lower *k* to 10 so that it correlates to a DNA based tool with *k* = 30. Figure 7 shows that kASA outperforms both tools in this scenario as the mutation percentage increases. However, for <6% mutations per read length the score is slightly lower than that of Kraken showing the information loss of the abstraction. This loss of precision is mitigated when *k* is increased which is the reason we use both the encoding and the range of *k*’s.

**Figure 8.**
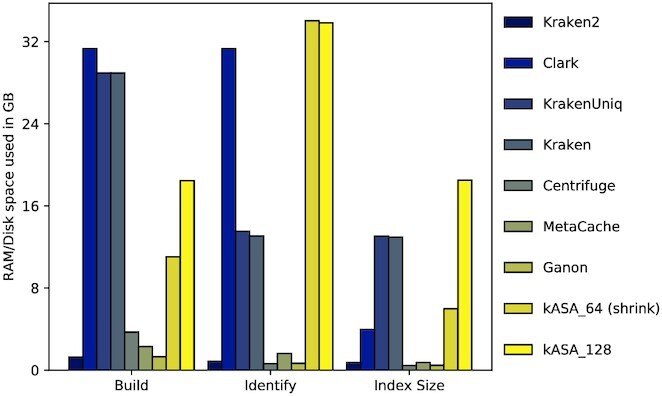
Amount of memory which is used to build the index or identify the test data, and sizes of indices for the robustness benchmark. Order is equivalent to the one in Figure 5. kASA is shown in with both integer sizes, for the 64 bit setting the index was shrunken in half via the lossless method.

We examined the memory consumption of each tool during index building and identification, as well as its index size (see Figure 8). Results show that with standard settings, kASA, Clark and Kraken/-Uniq have the highest memory and space usage. Please note that kASA has such high values because it allocates all available memory given (40GB in this case) during identification. In ‘build’, the memory usage will not exceed either the given restriction or the index size. Kraken2, Centrifuge, MetaCache and Ganon have a very low memory footprint in primary as well as in secondary memory in this benchmark due to our relatively small database.

**Figure 9.**
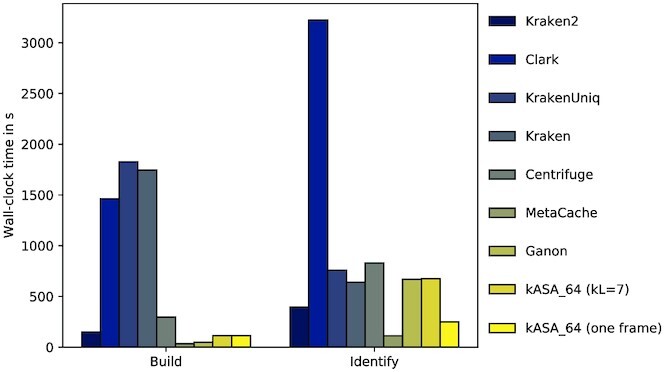
Wall-clock time in seconds for the tests. Times were gathered with the benchmark option in snakemake and sorted in the same manner as in Figure 5. Detailed measurements can be found in supplementary file C.

In terms of speed, Figure 9 shows that kASA is one of the faster tools for index creation and is as fast as Ganon for identification, being the middle field. Using only one frame makes it the second fastest tool for this benchmark. The speed of all tools is mainly due to the index fitting into primary memory. For the CAMI benchmark, where more data needs to be processed, we tested how kASA performs with different settings and hardware (see Figure 13). Further wall-clock times, e.g., with different lower *k*’s can be found in supplementary file C.

### Taxonomic profiling of simulated data

A taxonomic profile is a list of all organisms that have been identified in a dataset together with their respective abundance. This abundance is estimated by normalizing the relative frequency against genome length and ploidy. Consistent with other studies (e.g. (29)), we do not use this normalization for the following study. Instead, we count all matched *k*-mers per *k* and taxon and derive the relative frequencies as described in the methods section. All mentioned tools except kASA calculate the relative frequencies from the taxa reported per read. Note, that the non-unique relative frequency correlates to the approach of the other tools and is therefore chosen for a comparative basis. Results for the other relative frequencies can be seen in supplementary file D.

The CAMI-Challenge (25) measures accuracy not only by difference from a gold standard but also by correctly identified taxa at every level. Combined with OPAL (26) it creates a framework for profile testing. We chose data from the 2nd CAMI Toy Human Microbiome Project dataset (gastrointestinal tract) since it contains long (on average 2500 bases), noisy (Phred quality score of about 8) reads and a known gold standard profile with multiple large test files. Our pipeline downloads these files, creates indices based on the genomes used for the test data, and then profiles the 10 given test files. Afterwards, the profiles are converted into the CAMI format and OPAL creates an html file containing the results. After preliminary tests, we decided to include all tools from the first benchmark except Ganon since it could not produce any profiles due to too many errors inside the reads, and Krakenuniq which, having the same values as Kraken, seemed redundant. Because the test files contain long reads we chose the 128 bit setting for kASA and a lower *k* of 10. Therefore, our profiles contain columns for every *k* from 10 to 25. Furthermore, OPAL treats additional entries in the taxonomic profile as false positives even if their percentages are very low. To address this, we set different thresholds in the profiles on the species level where applicable. A threshold of 0.01 seemed to perform best while setting none at all performed worst regarding precision. We therefore recommend setting a cutoff value since any threshold performed better than none.

**Figure 10.**
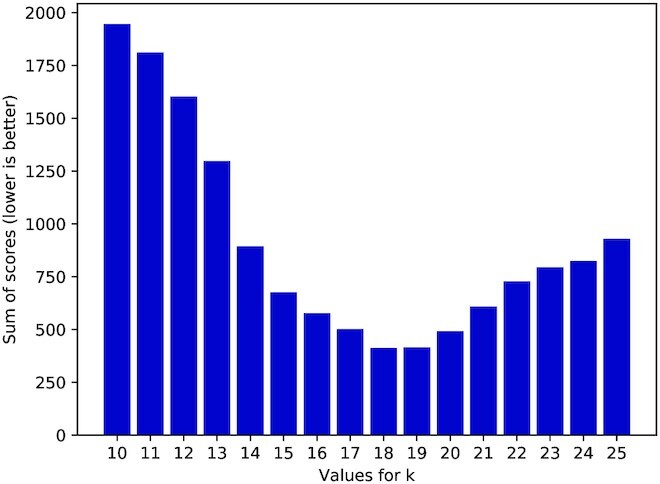
Plot showing the sum of scores given by OPAL for every *k* in the profile using the non-unique relative frequency with a cutoff of 0.01.

As seen in Figure 6 there is an interval of *k*’s performing superior than those before and after them. We therefore first tested which *k*’s performed best in this scenario. Figure 10 shows that *k*’s from 17 to 20 had the lowest and therefore best scores across all datasets. Note that for different data other *k*’s may perform better. There is no general rule which *k* to pick for every study which is the reason we include all *k*’s in our profiles. In most cases, using the largest *k* was sufficient. Therefore, we chose the *k*’s 18 and 25 for the following comparison with other tools.

Table 1 shows that kASA ranks first according to OPAL even if only one frame is used (due to better precision and L1 norm error) for *k* = 18. If the maximum *k* of 25 is used, the scores are still better than those of most tools. With normalization enabled in OPAL, Kraken ranks second and MetaCache (with threshold) third. We would like to mention that in the profiles of Kraken and Kraken2 taxa were often reported with an abundance of zero. If the normalization in OPAL is deactivated, both tools therefore drop in score considerably. Regarding the difference to the gold standard, all tools were able to come close to the true distribution (see beta diversity in supplementary file D). Overall, this indicates that kASA would perform quite well in a CAMI-Challenge given the opportunity to discard entries with low frequency.

**Table 1.** Sum of scores (S.o.s.) with and without (-n) normalization given by OPAL (lower is better)

Tool	S.o.s.	S.o.s -n
kASA-18-0.01	408	430
kASA-18-one	415	437
Kraken	421	883
MetaCache-0.01	425	409
CLARK-0.01	542	508
kASA-25-0.01	649	595
kASA-25	751	687
Kraken2	882	1351
MetaCache	887	865
Centrifuge-0.01	977	945
CLARK	1106	1063
Centrifuge	1463	1292

**Figure 11.**
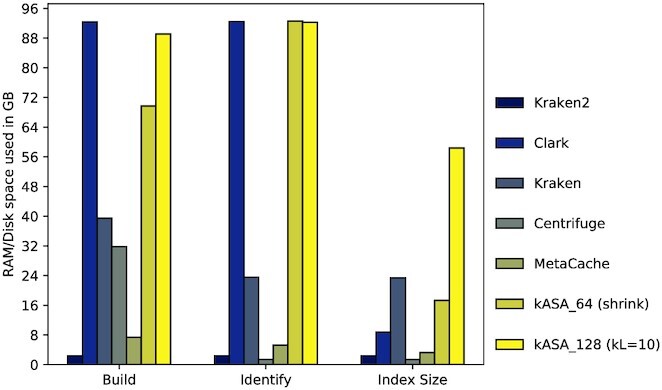
Amount of memory which is used to build the index or identify the test data, and sizes of indices for the CAMI benchmark. Order is equivalent to the one in Figure 5. kASA is shown with both integer sizes.

As done in the previous benchmark we measured memory usage and wall-clock time. Memory usage as seen in Figure 11 shows a similar pattern as in Figure 8 where kASA and Clark allocate as much memory as possible and given (100 GB in this case). We also see that Centrifuge now uses almost as much primary memory during building as Kraken due to the larger database. Kraken2 and MetaCache still use far less primary and secondary memory than the other tools but this comes at the cost of reduced robustness as seen in Figure 5. We would like to mention that should the database further increase in size those tools can deal with low memory requirements as well. Kraken2 has a parameter limiting the index size but sacrificing sensitivity. MetaCache and Ganon have the ability to partition their index into small enough parts and then sample iteratively against these indices. This has disadvantages, for example, a loss of cache efficiency or keeping track of multiple outputs. For the sake of completeness, we would like to mention that other mentioned tools not included in the benchmark have strategies to cope with memory restrictions as well. Unfortunately, this often either influences wall-clock times by a large degree or reduces accuracy. Centrifuge on the other hand exits with an error and the suggestion to run it on a system with larger primary memory.

**Figure 12.**
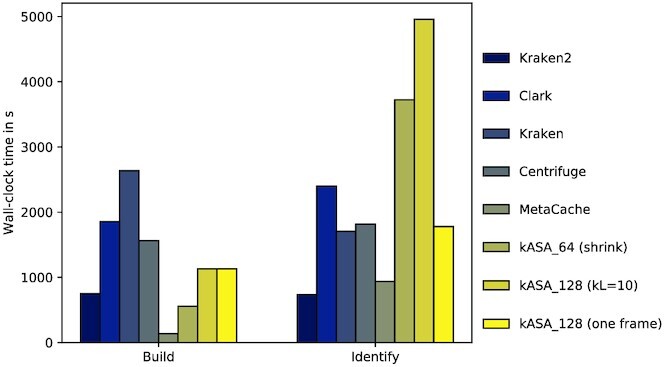
Wall-clock time in seconds for the CAMI datasets. Times were gathered with the benchmark option in snakemake and sorted in the same manner as in Figure 5.

Figure 12 also shows a similar trend as in Figure 9 although kASA now takes much more time than Clark, which is due to the input files being quite large (about 10GB each). When choosing to only process one frame instead of three, the time needed is significantly reduced.

**Figure 13.**
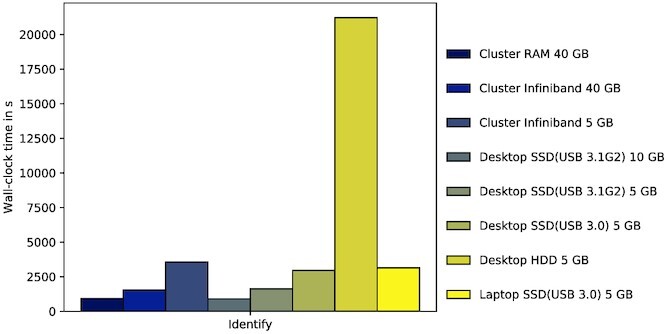
Wall-clock time in seconds for different platforms and settings. The legend must be read as following: Platform, hardware on which the index lies and interface technology, amount of RAM given. Details like the system specifications can be found in supplementary file A.

Lastly, we tried different settings and platforms for the 64 bit index of kASA and one input file of this benchmark. Figure 13 shows how kASA would perform on all mentioned platforms and with different locations of the index as well as memory restrictions. The first conclusion is that we do not recommend using an HDD since its hardware is not designed for the algorithm kASA uses to search for matches inside its index. We can also see that the interface technology and thus the throughput plays a big role since running this test on a desktop computer together with an SSD connected via USB 3.1 Gen 2 is almost as fast as using it in RAM on the cluster, even if less primary memory is available. Further evidence of this is the downgrade of the USB standard from 3.1G2 to 3.0 on our desktop which also had a visible impact. Finally, the CPU’s clock rate only has a minor impact since the wall-clock time on our laptop (2.3 GHz) is only slightly higher than that of our desktop (4.5 GHz).

### Analysis of real data

To test kASA’s ability to identify known organisms in real data, we used data sampled from human saliva (Sample ID: SRS147126, SRA ID: SRX154235) from the Human Microbiome Project (30) to create taxonomic profiles and compare the results with those created by Kraken2 and Centrifuge equipped with the same database and in case of Kraken2 additionally with the Minikraken index (8GB in size). We chose the default version of kASA with a maximum *k* of 12 because reads were of length 100 bases. The database was created with all genomes from the Refseq database as of 2019-10-01. This database (ca 1.1TB in size) resulted in an index of 1.4TB in size for kASA after shrinking by 50% during building and then lossless in half afterwards. The size of the index seems to be quite large but given the fact that external SSDs of 2TB size are becoming more widespread and affordable, we would argue that this does not affect the usability of kASA. Nevertheless, we created an additional index with a Laptop of 8GB RAM equipped with such a 2TB external SSD (connected via USB 3.0) containing all bacteria from the Refseq (date of file retrieval: 2020-12-09) to give a perspective how long such study would take. Building while using the same procedure as for the complete RefSeq index took one and a half day and resulted in an index the size of 264GB, identification took about 23 hours (using six translation frames and 7GB of primary memory). The resulting relative frequencies are close to those calculated with the larger index (Pearson correlation coefficient of >0.95).

Table 2 shows that all tools agree in the most abundant genera although in different proportions and in case of kASA, depending on unique or non-unique relative frequency. All detected genera are known to occur in the human oral flora which confirms our results. The complete tables are shown in supplementary file E. Supplementary file F contains a visualization of all identified species via Krona (31).

**Table 2.** Selection of genera with the highest relative frequency (in %) found in data sampled from human saliva (SRS147126). Results for kASA are shown with unique and non-unique values for *k*= 12

Scientific Name	kASA u.	kASA n.-u.	Kraken2	Centrifuge	Kraken2 + Minikraken
Prevotella	43.5	27.9	30.7	34.1	5.1
Streptococcus	9.9	16.5	13.5	17.8	3.1
Neisseria	4.8	15.2	11.5	9.1	2.7
Veillonella	7.8	7.7	7.7	6.9	0.6
Porphyromonas	9.2	6.6	6.8	0.3	0.02
Haemophilus	3.9	5.1	4.5	3.8	1.0
Actinomyces	2.2	1.7	2.1	1.1	0.2
Campylobacter	2.8	1.6	1.4	1.1	0.3

## DISCUSSION AND CONCLUSION

kASA enables taxonomic identification and profiling of metagenomic data even if hardware resources are limited. In addition, it uses two approaches to increase robustness against erroneous bases in reads: An amino acid-like encoding and a range of *k*’s for its *k*-mers. We created two benchmarks to test the behavior for both analyses and with this, the validity of both approaches combined and by themselves.

The identification benchmark shows that our tool has a better robustness against sequence errors than all other presented tools. It also shows that the range of *k*’s has a direct influence on the robustness, even more than the amino acid-like encoding (see Figures 6 and 7). Using three or more frames increases robustness even further (see Figure 5) but even if only one frame is used, we still reach higher scores than the other tools and at the same time reduce wall-clock times significantly.

The profiling benchmark supports these observations as input data used for this test was highly erroneous. There, kASA ranked highest as well, even if only one frame is used. Choosing not the optimal but the maximum *k* still results in good scores. While this may tempt users to always use the highest *k*, as we have done in the real data experiment in Table 2, we recommend looking at the whole profile or calculating the distribution from the identification file (as most other tools do). Otherwise, species that have a high unique relative frequency for lower *k*’s than the maximum one might be missed.

Regarding algorithmic efficiency, our focus was to optimize accuracy and wall-clock times in a primary memory restricted environment. Therefore the secondary memory usage is higher in our standard version which uses much more than other tools, especially when *k* is higher than 12 (see Figures 8 and 11). However, we provide several methods to balance wall-clock times, secondary memory usage and primary memory consumption. If users need a smaller index, they can reduce the index either with or without information loss (if the requirements are met) and use one or three frames instead of six during build. This may reduce accuracy. On the other hand, if wall-clock time is critical, users can use more resources or reduce the number of frames and load the index into RAM, which consequently leads to greater consumption of primary memory. Finally, if primary memory is limited, users can set a limit that kASA does not exceed and the index can be moved to an SSD, for example. This increases wall-clock times (see Figure 13).

We conclude, that the use of a range of *k*’s instead of a fixed word length proves to be a valid method for improving sensitivity without sacrificing precision as long as the lower bound of *k* is reasonably high (we recommend a value of at least 5). The abstraction to an amino acid-like encoding further increases the robustness of kASA to mutations without using gapped sequence alignment. This shows that our approach can compete with or even exceed commonly used approaches.

In addition, kASA is, to our knowledge, the only available tool providing a RAM consumption so highly adjustable that it also works on cheaper computers at reasonable speed and with superior accuracy. Because secondary memory is much cheaper than primary memory and external SSDs are becoming more widespread, virtually every life scientist should now have the technical means to analyze metagenomic data in depth.

## DATA AVAILABILITY

kASA is freely available on https://github.com/SilvioWeging/kASA and licensed under Boost Version 1.0. The Snakemake pipelines are available on https://github.com/SilvioWeging/kASA_snakemake and https://github.com/SilvioWeging/kASA_cami.

## Supplementary Material

gkab200_Supplemental_Files
