# Supplementary material for: Taxonomic analysis of metagenomic data with kASA
Source: Nucleic Acids Res. 2021 Mar 30;49(12):e68. doi: 10.1093/nar/gkab200 (PMC8266618; doi:10.1093/nar/gkab200)
Supplement: gkab200_Supplemental_Files [file gkab200_supplemental_files.zip › Suppl_A.pdf]

Supplemental file 1 for kASA

Silvio Weging

February 12, 2021

Contents

1 Evaluation of synthetic data **2**
1.1 Formulas 2
1.2 Further results 4

2 Further experiments **11**
2.1 McIntyre et. al. 11
2.2 Lindgreen et. al. 12
2.3 Shrink 15
2.4 Kaiju 16

3 Pseudocode of the identification algorithm **19**

4 Versions and system specifications **24**

5 Information loss of the amino acid-like encoding **25**
5.1 Experiments with different alphabets 27

1 Evaluation of synthetic data

1.1 Formulas

We evaluated the identification quality for our synthetic tests from two perspectives: Read and Taxon.

The former is done by checking whether the original taxon of a read was identified correctly by ID. If the ID matched the one defined for that read, it was marked as a correctly identified and assigned read, otherwise only as assigned (decreasing both sensitivity and precision). Only the best hits were considered. For kASA the json array for every read containing the "Top hits" was used. This array is calculated by normalization of the k-mer scores to $[0, 1]$ and including everything with a normalized value ≥ 0.8. This value seemed to correspond best to what would intuitively be considered "relevant" when reporting several results for one read. If two or more taxonomic IDs matched the read or the LCA-based algorithms gave a higher taxonomic rank as result (while containing the correct ID), the read was additionally considered ambiguous but correctly assigned. If the taxonomic path given by backtracking the LCA-path did not contain the correct ID, it was considered incorrectly assigned. We added genomic reads from species not inside the database/indices to test every tool's ability to "ignore" reads. This is measured with the specificity via checking if a nonassignable read was correctly not assigned. The formulas are as follows:

$$\text{Sensitivity} := \frac{|\text{Correctly assigned reads}|}{|\text{Reads}|}$$

$$\text{Precision} := \frac{|\text{Correctly assigned reads}|}{|\text{Assigned Reads}|}$$

$$\text{F1 score} := 2 \cdot \frac{\text{Sensitivity} \cdot \text{Precision}}{\text{Sensitivity} + \text{Precision}}$$

$$\text{Specificity} := \frac{|\text{Correctly unassigned reads}|}{|\text{Nonassignable Reads}|}$$

Because of this setup, tools reporting everything from their index with the same score would get an artificially high F1 score. To counter this, we added the perspective of each taxon which can be done via a binary classification. The "original read taxon" is the one we know, the "reported taxon" is the one the tool returns for that read.

- True positives (TP) - The original read taxon did expect the reported taxon and got it.
- True negatives (TN) - The original read taxon did not expect the reported taxon and it was (correctly) not reported.
- False positives (FP) - The original read taxon did not expect the reported taxon but got it anyway.
- False negatives (FN) - The original read taxon did expect a taxon but there was none reported.

With this, we can calculate the four values for every expected taxon and derive the Matthews correlation coefficient:

$$\text{MCC} := \frac{(TP * TN - FP * FN)}{\sqrt{(TP + FP) * (TP + FN) * (TN + FP) * (TN + FN)}}$$

We then averaged these MCCs for every file and got our measure how often a tool reports only what is necessary.

The evaluation was performed by a script for each output, because no standard for tool outputs exists. Mutations include insertions, deletions and single point mutations, all with the same probability. Indices where these mutations occur per read are drawn randomly via Python. The seed is fixed for recreating data. Read length was fixed to 100 and per genome, $\frac{|\text{bases}|}{100}$ reads were generated. Positions in the genome from which the reads were sampled were also drawn randomly.

Please see our GitHub site for further details on how to recreate our results: `https://github.com/SilvioWeging/kASA_snakemake`.

1.2 Further results

The following figures show the results of our robustness benchmark for all lower k's from 7 to 12 for kASA, the MCC for the tested tools, and the F1 score for all tools and k's from 7 to 12.

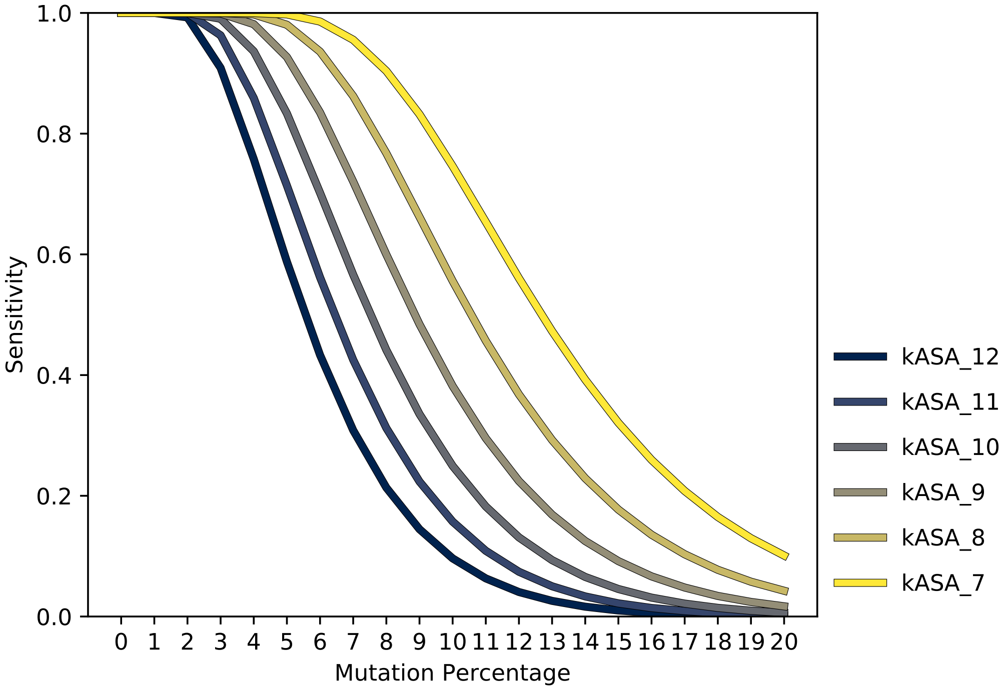
1.0
0.8
0.6
0.4
0.2
0.0
Sensitivity
0 1 2 3 4 5 6 7 8 9 10 11 12 13 14 15 16 17 18 19 20
Mutation Percentage
kASA_12
kASA_11
kASA_10
kASA_9
kASA_8
kASA_7

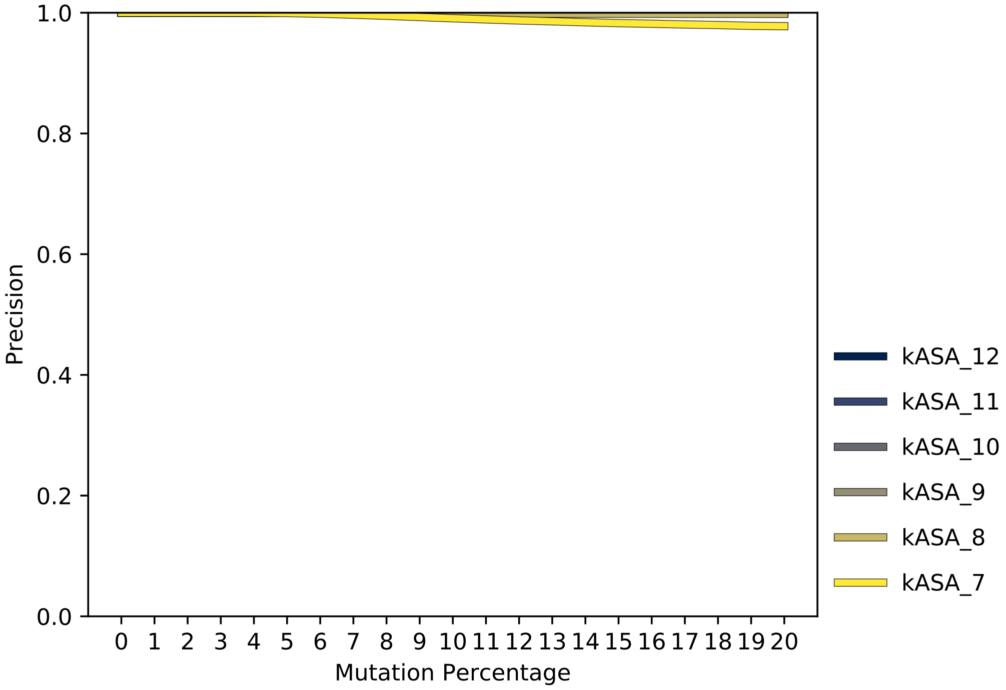
1.0
0.8
0.6
0.4
0.2
0.0
Precision
0 1 2 3 4 5 6 7 8 9 10 11 12 13 14 15 16 17 18 19 20
Mutation Percentage
kASA_12
kASA_11
kASA_10
kASA_9
kASA_8
kASA_7

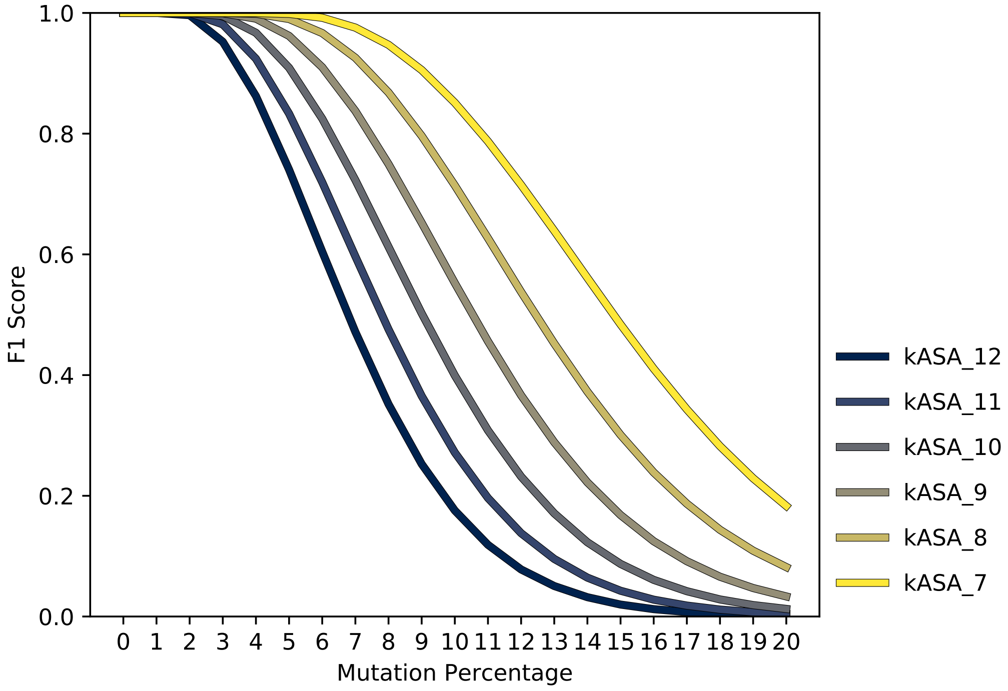
F1 Score
Mutation Percentage
kASA_12
kASA_11
kASA_10
kASA_9
kASA_8
kASA_7

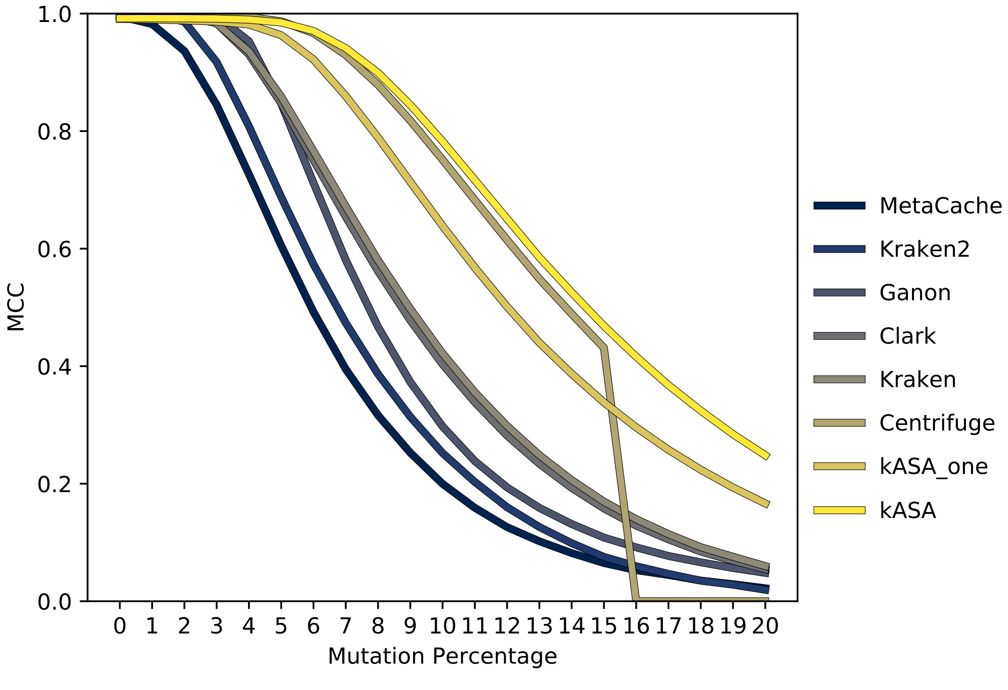
MCC
Mutation Percentage
MetaCache
Kraken2
Ganon
Clark
Kraken
Centrifuge
kASA_one
kASA

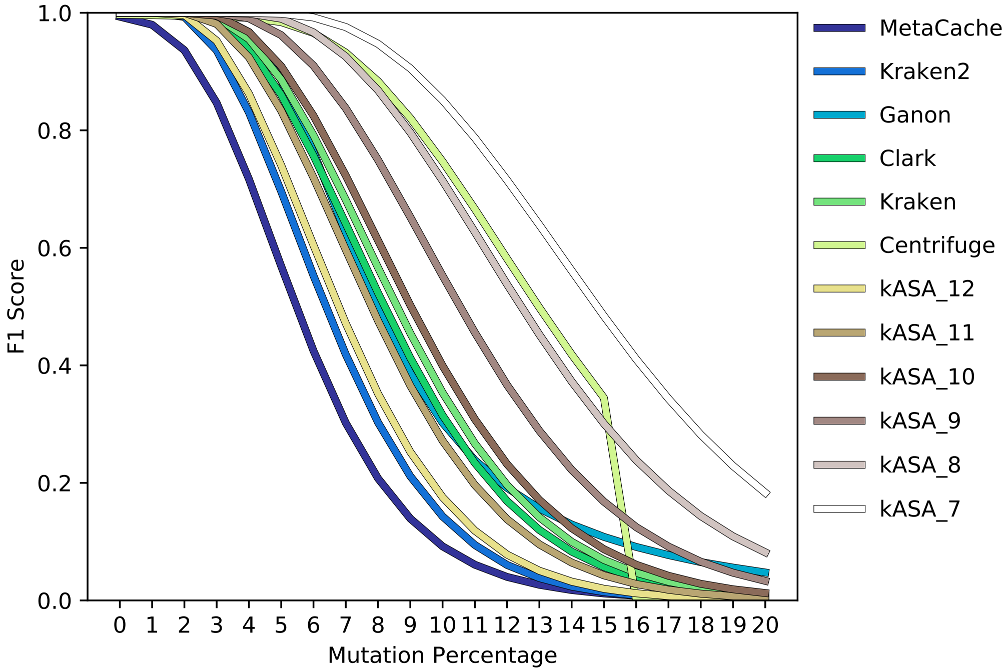

1.0
0.8
0.6
0.4
0.2
0.0
F1 Score
0 1 2 3 4 5 6 7 8 9 10 11 12 13 14 15 16 17 18 19 20
Mutation Percentage
MetaCache
Kraken2
Ganon
Clark
Kraken
Centrifuge
kASA_12
kASA_11
kASA_10
kASA_9
kASA_8
kASA_7

Since for a lower k of seven, kASA performs best in the above figures, we tested how the specificity is affected by our approach. The next figures show that it is inversely correlated with mutation percentage. This is due to short similarities with negatives in our benchmark. However, if a threshold is applied, the specificity returns to acceptable values. For the test data with zero mutations, a threshold of 0.4 on the relative score was able to yield almost perfect specificity without lowering sensitivity or precision. However for data sets with a higher number of mutations, the ROC is still acceptable but the sensitivity suffers visibly (Dots mark results and numbers are the applied thresholds).

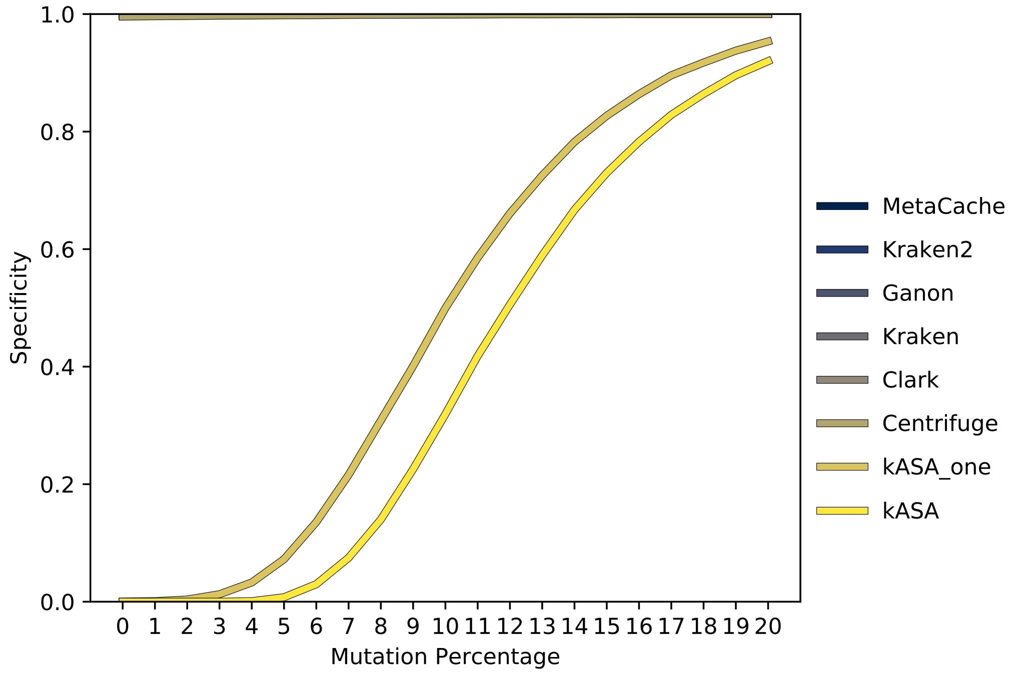

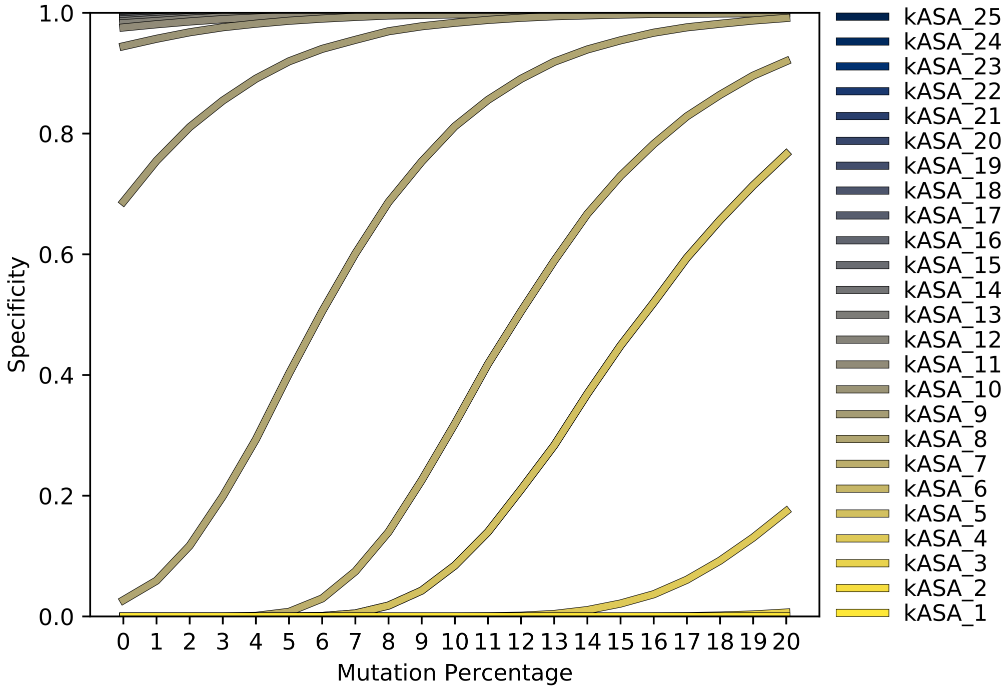
1.0
0.8
0.6
0.4
0.2
0.0
Specificity
0 1 2 3 4 5 6 7 8 9 10 11 12 13 14 15 16 17 18 19 20
Mutation Percentage
kASA_25
kASA_24
kASA_23
kASA_22
kASA_21
kASA_20
kASA_19
kASA_18
kASA_17
kASA_16
kASA_15
kASA_14
kASA_13
kASA_12
kASA_11
kASA_10
kASA_9
kASA_8
kASA_7
kASA_6
kASA_5
kASA_4
kASA_3
kASA_2
kASA_1

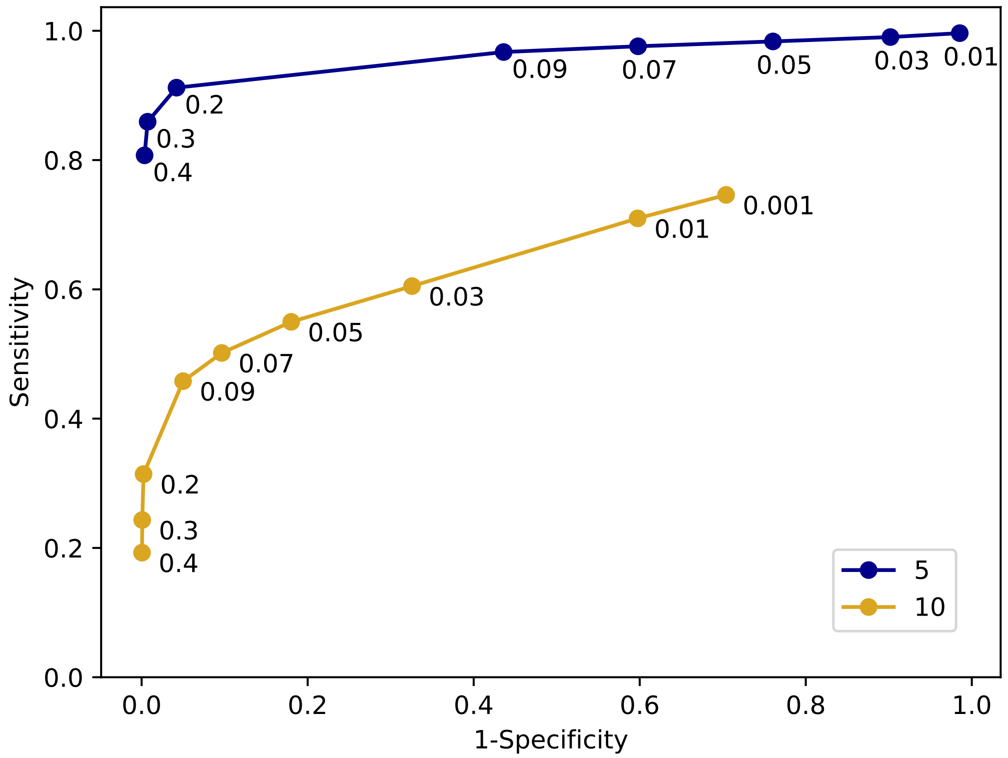
1.0
0.8
0.6
0.4
0.2
0.0
Sensitivity
0.0 0.2 0.4 0.6 0.8 1.0
1-Specificity
0.09
0.07
0.05
0.03
0.01
0.2
0.3
0.4
0.001
0.01
0.03
0.05
0.07
0.09
0.2
0.3
0.4
5
10

We also wrote that a lower k of seven was set as default to fully reduce the search space inside the index. From there on, the number of k's that need to be checked, influences the execution time with linear time complexity. The following figure shows this very clearly. It was generated by taking one file from the CAMI snakemake benchmark and then measuring the execution time of different values for the lower k.

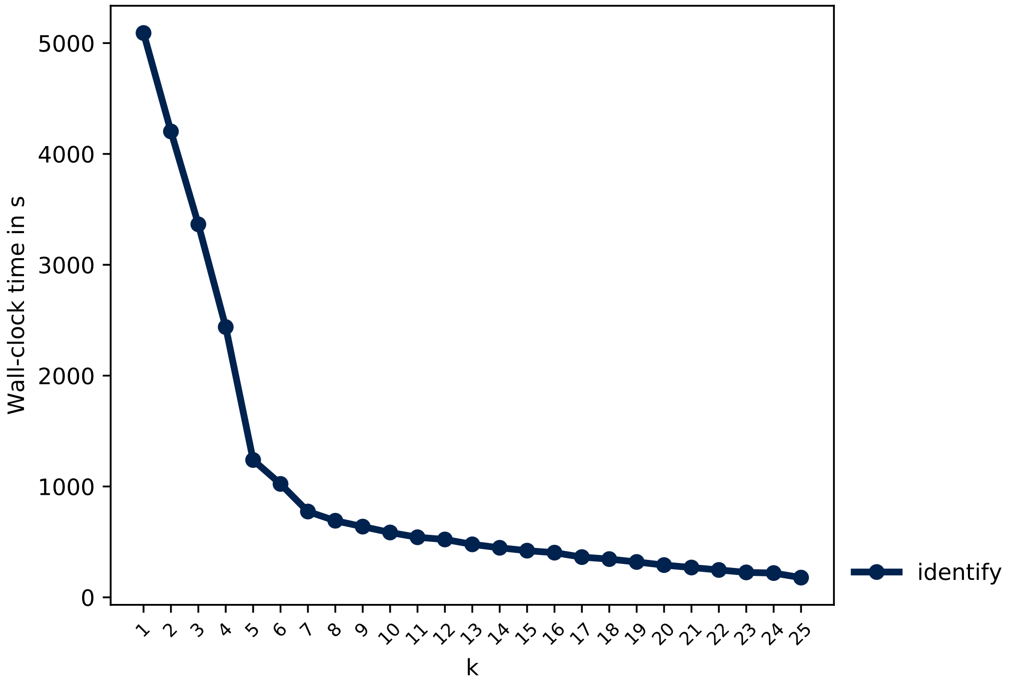

2 Further experiments

We also compared the performance of kASA with existing benchmark data. Since this data is static, as are the tools used (and thus the results may become stale over time), we did not include these benchmarks in the main manuscript, yet we thought it to be of interest to some users.

2.1 McIntyre et. al.

The study of McIntyre et. al.[1] was primarily designed to test, how false positives during a metagenomic study can be reduced. This included running combinations of tools on the same data to see if it would increase confidence (and thus reduce false positives) in a result to, e.g., make clinical decisions based on the composition of the sample. As a ground truth, multiple data sets were designed and multiple tools ran on their own and in combination (e.g., BLAST and MEGAN). Of the 35 data sets in this study we selected 10 simulated ones: two files with high complexity and even distribution of species (HC) and eight files with low complexity and log-normal distribution of species (LC). This selection was made based on accessibility of the ground truth which is contained in the headers of every read for these data sets, making the evaluation simple. The used quality measures of precision and sensitivity and the resulting F1 score are the same above. We chose the taxonomic level "species" because it strikes a balance between index size (the lower the taxonomic level, the larger the index) and complexity. The publication offered results for subspecies, species and genus level so we did not have to rerun other tools and could just add results for kASA to the table. The Figure below shows the median of the F1 scores from the results for the selected data sets. For kASA we had to choose two different methods for the evaluation. The first is similar to how tools that do not support multiple assignments per read operate and take only the taxon with the highest score for every read (thus named kASA_first). The second takes all taxa with almost the same score as the best one into consideration, which is equivalent to printing out multiple assignments per read (named kASA_all).

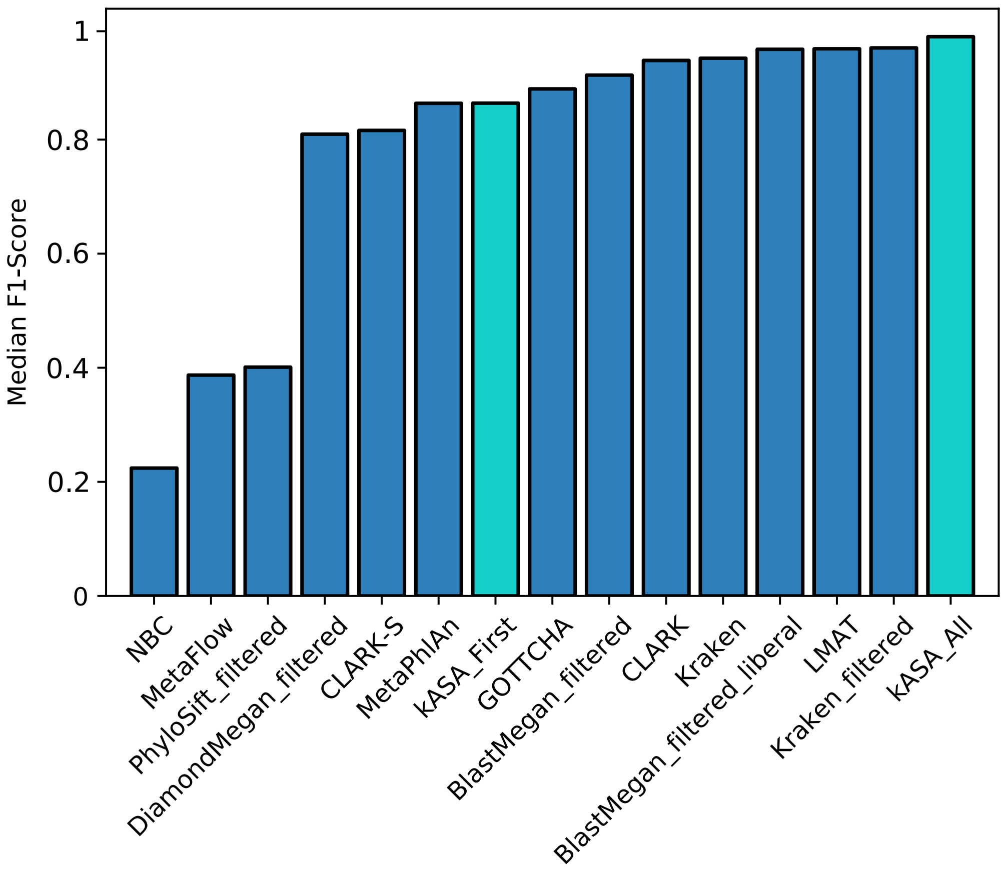

Using only the first best taxon for every read results in a sufficiently high F1 score in comparison to other tools. However, the real strength of kASA is the ability to print out multiple assignments per read with an equal or slightly lower score. This leaves the user with more information to work with and we see that it results in the best F1 score of all tools.

2.2 Lindgreen et. al.

We also evaluated kASA with the same data, methods and quality measures as used in a metastudy by Lindgreen et. al. [2]. Although we did not run any of the tools studied in the paper except Clark again, we recalculated all measurements to ensure consistency.

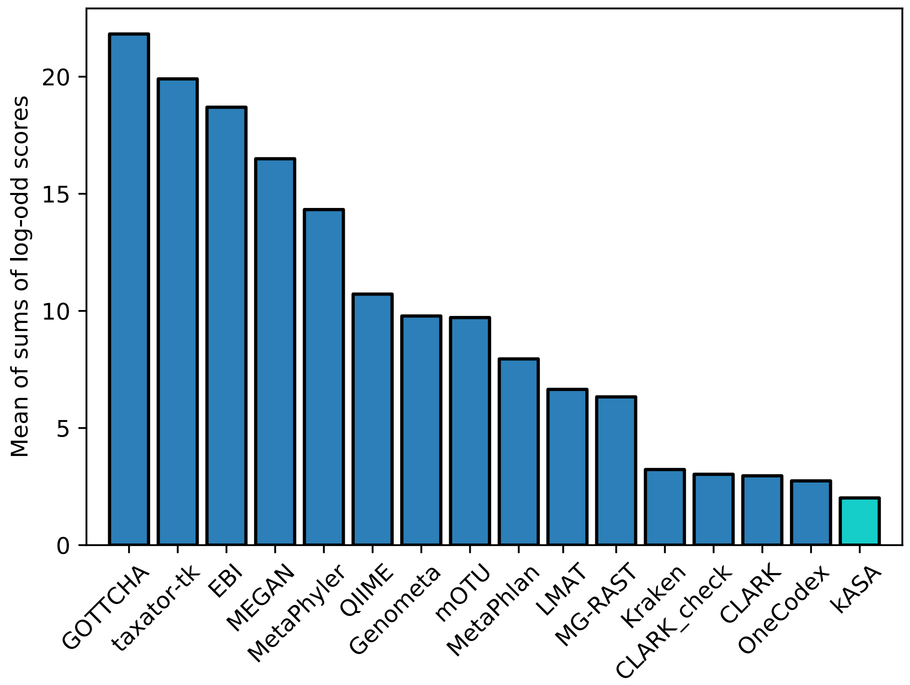

Figure 1: Profile quality measured by the mean of log-odd scores. The log-odds of absolute differences between relative frequency and gold standard were summed per dataset and averaged regarding all six datasets. Tools are sorted by this score meaning lower is better. Data from the corresponding publication [2] was also used in the publication of Centrifuge [3] where the authors wrote, that their accuracy was similar to that of Kraken.

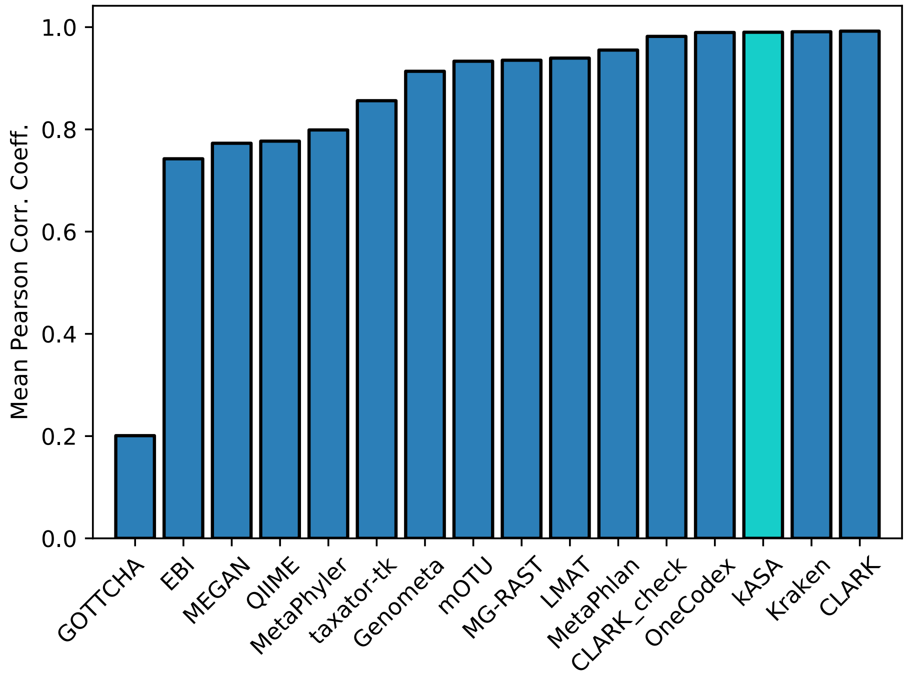

Figure 2: Profile quality measured by the mean of Pearson Correlation Coefficients. The Pearson Correlation Coefficients of vectors of relative frequencies in relation to the respective truth for every dataset were averaged and shown here. Tools are sorted by that value. Results for the best four tools differ only slightly.

Figure 1 and 2 show that kASA is the best tool regarding log-odd scores and only slightly differs in value for the Pearson correlation coefficient. Relative frequencies for this comparison were gathered per read for consistency, as mentioned above, but profiles generated by a direct use of k-mer frequencies deviate only slightly.

2.3 Shrink

In the main manuscript we mentioned that an index can be shrunken to fit a certain size. One method is to assume that not more than $2^{16} - 1 = 65535$ entries are inside the content file and that $k \in [7, 12]$. This ensures that the prefix trie contains all 6-mers of the index and thus half of the letters of every k-mer from a 64-bit index. This half is then deleted which halves the index size without losing information since the prefixes are still inside the prefix trie. Another method is to delete k-mers from the index. Either a target size is given or a percentage. This calculates, based on the frequency file containing how many k-mers for every taxon are inside the index, the number of k-mers which need to be deleted for every taxon. Meaning that a taxon with more k-mers loses more entries than one with fewer k-mers. We considered this fair since e.g. a virus needs to keep more k-mers to still be detectable than the human genome. The figure below shows the effect on the robustness should a given percentage be kept (aside from that, default settings were used). kASA-100 is the full index, kASA-90 only 90% of this index and so on. We see that the impact is measurable but not significant except for 10% and 20%. This effect was used for the real data experiment were we shrunk the index by about 60% first and then halved it with the first method.

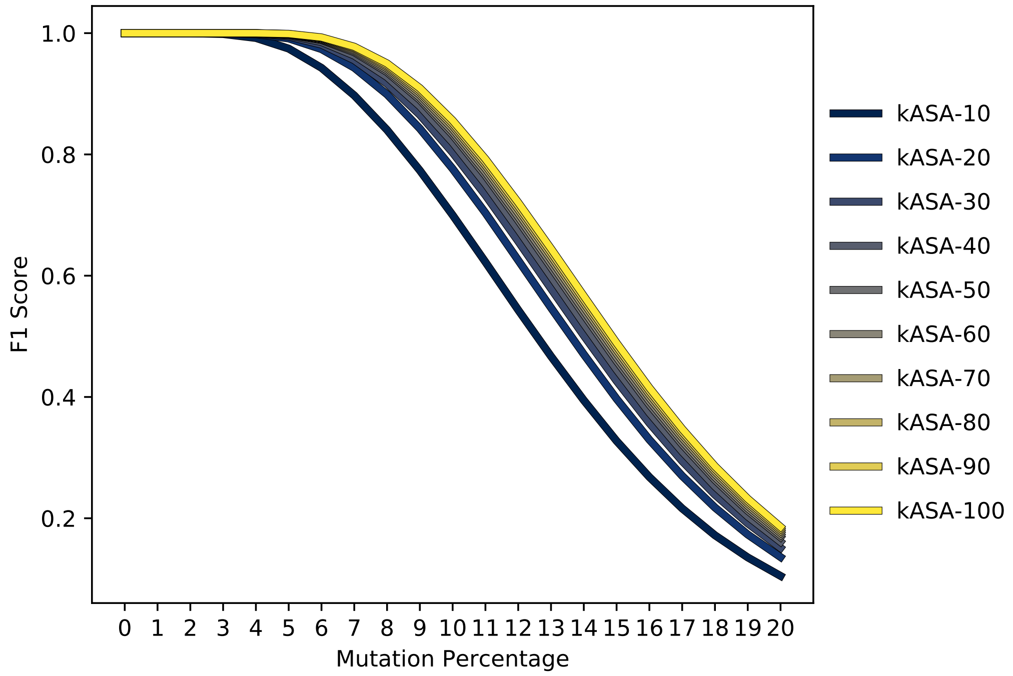

2.4 Kaiju

Kaiju [4] is a Burrows-Wheeler transform [5] based tool using an FM-index [6] and needs protein sequences as database for its index. We have refrained from including it into both of our benchmark pipelines since its database is not based on nucleotide sequences/genomes. But since kASA is able to use protein sequences as database for its index as well, we could compare both tools head to head. For this we used the "Fungi" database from the website of Kaiju containing gbffs with genomes as well as translated sequences (genes etc.) of various taxa (the bacterial database was deemed to large for this small experiment). The translated sequences served as a database from which we created the indices. We also created mutated sequences from the genomes since Kaiju allows nucleotide sequences as input and therefore we proceeded like in our robustness benchmark (albeit with fewer mutation percentages). Since the authors wrote that Kaiju benefits from longer reads we sampled 200 bps instead of 100 bps (as done in our robustness benchmark) from the genomes randomly. For kASA we used the '–six' flag to indicate that translation of the input needs to occur in six frames since our index consists of already translated sequences without any indication of direction. Since genomes of fungi contain non-coding regions, we do not expect both tools to be able to identify every read. This is the main disadvantage of using only coding regions and their translated sequences as database - something

we think is the biggest difference between the two tools aside from their identification method.

Technical details and settings (platform was HPCC): Kaiju version 1.7.4, 5 cores used during build, 5 GB RAM used (30 GB given) for build, 2.8 GB resulting index size, 5 min needed for build, 8 cores used during identification, about 3 GB used for identification, about 7 min in total used for identification. kASA version 1.4, 5 cores during build, 19 GB RAM used (30 GB given), 18 GB resulting index size, 6 min needed for build, "species" as taxonomic level used, 8 cores given for identification, 28 GB RAM used (30 GB given, index loaded into RAM), `identify_multiple` used and about 13 min needed.

Results showed that by default, kASA has a higher sensitivity as well as a better robustness than Kaiju. Precision for kASA was lower (for zero mutations: 0.22 vs 0.73) because very low scoring hits were also reported. To be more concrete: True positives usually had k-mer scores of more than 15, false positives less than 1. We could therefore apply a threshold and improve precision (everything with a k-mer score lower than 1 was discarded). However this threshold would only apply to this data. The exact values can be found in supplementary file B. We are confident that any user looking at the identification output would be able to distinguish between a true hit and one with short, low scoring similarities.

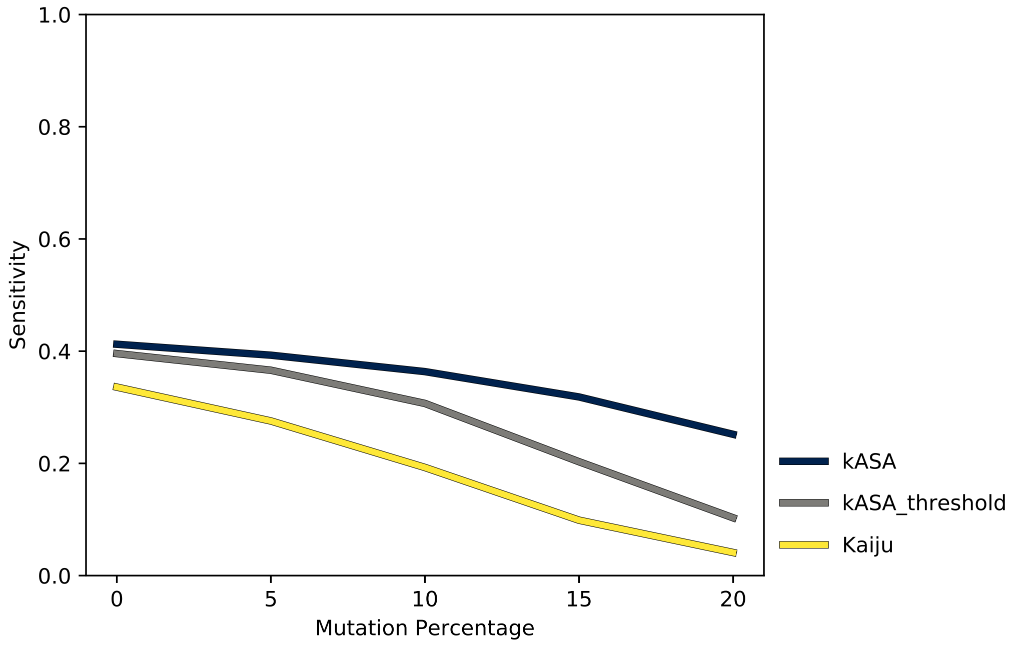

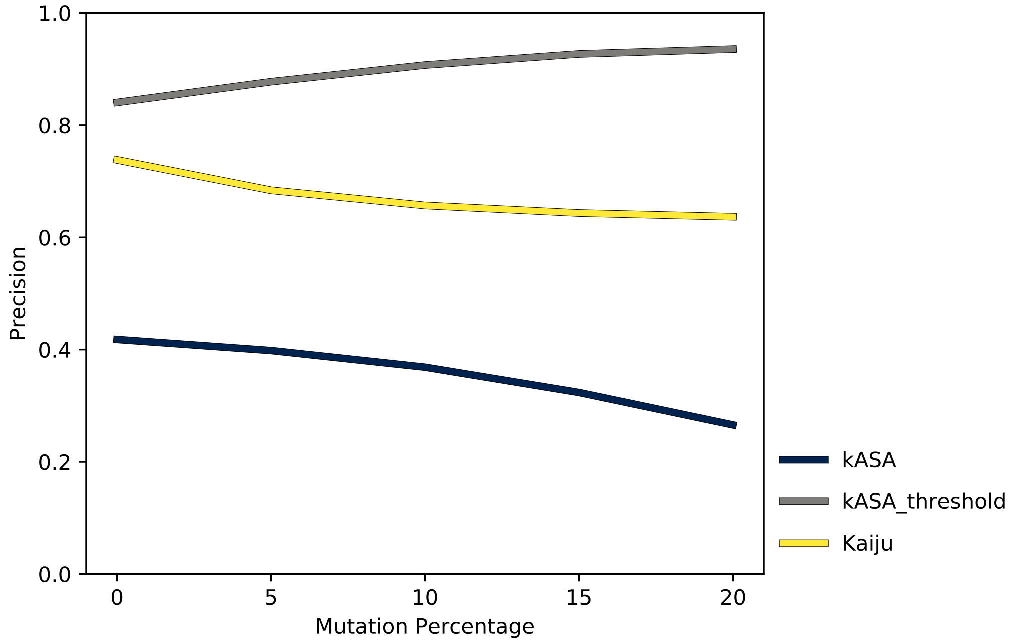
1.0
0.8
0.6
0.4
0.2
0.0
Precision
0
5
10
15
20
Mutation Percentage
kASA
kASA_threshold
Kaiju

3 Pseudocode of the identification algorithm

Customised set intersection algorithm used for a comparison of NGS data with the index. It computes both the profile and the identification file per read. After the pseudocode, an example is shown in Figure 3 which displays the workings of the algorithm.

Algorithm 1: Identify

```
input  : The index and the sorted input with converted k-mers and
         ranges
output: Scores and counts
// Part One
list of matched read IDs per k: rIDs_k ← [];
list of matched tax IDs per k: tIDs_k ← [];
list of last known k-mer per k: known_k ← [];
for Every entry with range in input do
    reset all lists;
    k-mers[lower,upper] = gather all k-mers with the same range;
    for All k-mers in k-mers[lower,upper] do
        x ← current k-mer;
        if range invalid then
            continue;
        currKMerShifted ← x with lowest value for k;
        start ← lower;
        end ← upper;
        do once
            if currKMerShifted is in range then
                use binary search to find the start;
            else
                continue;
        if x has been seen before then
            for all k's do
                add read ID to rIDs_k if x_k matches entry in known_k;
            continue;
        else
            seen before ← x;
        // Part Two
        // see below
    // Part Three
    // see below
return Scores and counts
```

```
// Part One
// see above
for Every entry with range in input do
    for All k-mers in k-mers_[lower,upper] do
        // Part Two
        for y in index from start to end do
            for all k's from lowest to highest do
                x_k ← x shortened for this k;
                y_k ← y shortened for this k;
                if x_k < y_k then
                    for all remaining k's from k to highest do
                        add read ID to rIDs_k if x_k matches entry in
                          known_k but avoid duplicates;
                    break out of the y loop and get next x;
                else
                    if x_k == y_k then
                        if x_k matches entry in known_k then
                            add tax ID from y to tIDs_k and read ID to
                              rIDs_k but avoid duplicates;
                        else
                            // all possible matches for old x_k
                               found -> save
                            for all entries in tIDs_k do
                                count unique or non-unique match;
                                for all entries in rIDs_k do
                                    save match of read ID and tax ID;
                                reset rIDs_k and add the current read
                                  ID;
                                reset tIDs_k and add the current tax ID;
                                known_k ← x_k;
                    else
                        // x_k > y_k
                        iterate y further through index and add tax IDs
                          to tIDs_k if y_k matches entry in known_k;
                        stop if no full match occurs;
                        break out of k loop;
            if highest k was reached then
                get next y;
        // Part Three
        // see below
```

```
for Every entry with range in input do
    for All k-mers in k-mers[lower,upper] do
        // Part One and two
        // &lt;see above&gt;
        // Part Three
        // look for any remaining y in case there are no
           more x left
        for any y left in range do
            for k from lowest to highest do
                if y_k matches entry in known_k then
                    add tax ID to tIDs_k;
                else
                    break;
            if at least one k matched then
                get next y;
            else
                break;
        // all possible matches for old x_k found -> save
        for all entries in tIDs_k do
            if size of tIDs_k == 1 then
                save unique match;
            else
                save non-unique match;
            for all entries in rIDs_k do
                save match of read ID and tax ID;
return Scores and counts
```

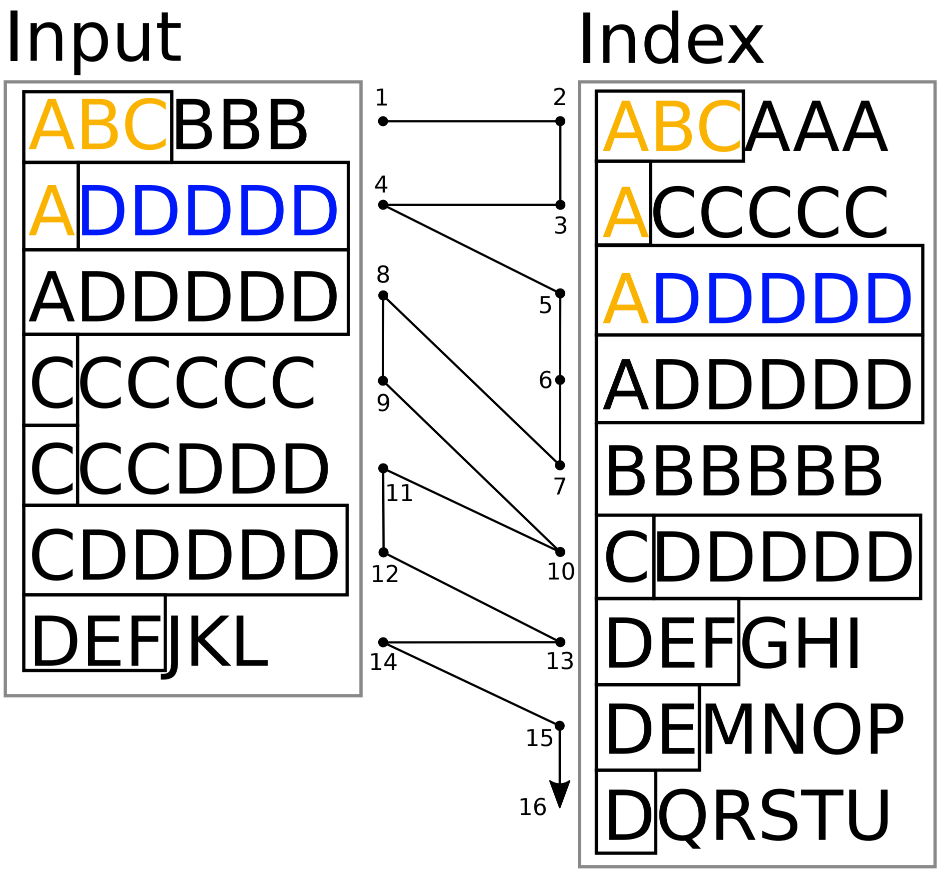

Figure 3: Schematic of the identification algorithm with a more complex example. The arrow in the middle together with the numbers show the order of execution. Rectangles around the letters mean matching letters. Color is used in the first k-mers to show how known k-mers are matched (step 3 matches with the "A" from step 2).

4 Versions and system specifications

Table 1: Versions of used tools.

Tool	Version or date
kASA	1.4 2020-11-19
Clark	1.2.5
Centrifuge	1.0.4 2020-10-02
Kraken2	2.0.8 2020-03-02
Kraken	1.1.1
KrakenUniq	0.5.8

HPCC - iDiv EVE

- DELL R640
- CPU: 2x 20-Core Intel(R) Xeon(R) Gold 6148 @ 2.40 GHz or Intel(R) Xeon(R) E5-2690 @ 2.9 GHz
- RAM: 1480GB DDR4 up to 2993MT/s
- Connected via InfiniBand(TM)

Desktop

- CPU: Intel(R) Core(TM) i7 7700K @ 4.5 GHz
- RAM: 16GB DDR4 - 2800 MHz
- SSD: Samsung 970 EVO(M.2)

Laptop

- CPU: Intel(R) Core(TM) i7-6500U @ 2.5 GHz
- RAM: 8GB DDR3 - 1600 MHz
- SSD: Samsung T7(USB 3.1G2, 2TB)

5 Information loss of the amino acid-like encoding

Let $\mathcal{A}$ be an alphabet of size n with $1 \leq n \leq 28$, $n \in \mathbb{N}$ and $\mathcal{B} := \{A, C, G, T\}$. Let furthermore S be a word consisting of at least three letters from $\mathcal{B}$, so $S \in \mathcal{B}^*$ and $|S| \geq 3$.

Let $\texttt{code} : \mathcal{B} \times \mathcal{B} \times \mathcal{B} \to \mathcal{A}$ be a function with the following property:

$$\texttt{code}((b_0, b_1, b_2)) = \texttt{code}((c_0, c_1, c_2)) \Rightarrow b_1 = c_1, \quad (b_0, b_1, b_2),\ (c_0, c_1, c_2) \in \mathcal{B} \times \mathcal{B} \times \mathcal{B}. \tag{$\star$}$$

$\texttt{translate} : \mathcal{B}^* \to \mathcal{A}^* \times \mathcal{A}^* \times \mathcal{A}^*$ is now a function for translating the DNA sequence S into three amino acid-like sequences with iterative application of $\texttt{code}$. If $\texttt{code}$ is applied with a shifted start, we get the conversion in three frames described in the paper. So the resulting words w_0, w_1, $w_2 \in \mathcal{A}^*$ are as follows:

$$w_j := \bigoplus_{i=j}^{\lfloor \frac{|S|-j}{3} \rfloor} \texttt{code}(S\,[j + 3 \cdot i, j + 3 \cdot i + 3]),\ j = 0, 1, 2$$

where $\bigoplus$ is the string concatenation.

Observation 5.0.1. *w_0, w_1, w_2 are created from overlapping triplets, which means that for a DNA sequence b_0, b_1, b_2, b_3, b_4, ... the triplet of the first frame b_0, b_1, b_2 shares two letters b_1, b_2 with the second frame and one letter b_2 with the third frame. Furthermore, the second frame b_1, b_2, b_3 shares b_2, b_3 with the third frame which starts with b_2, b_3, b_4. b_3 and b_4 are now again the bases forming the first and second letter in the first frame.*

Lemma 5.1. *Apart from the first and last letter of S, no information loss occurs when using* `translate`*.*

Proof. To show that no information loss occurs, except for the first and last letter of S, we construct an appropriate inverse function $\texttt{translate}^{-1}$. We assume without loss of generality, that $\{w_0,\ w_1,\ w_2\} \in \mathrm{im}(\texttt{translate})$.

$\texttt{code}^{-1}$ is created by determining each triplet $b \in \mathcal{B} \times \mathcal{B} \times \mathcal{B}$ associated with the respective letter $a \in \mathcal{A}$ and storing it in a dictionary with a as key. This means, that e.g. for $w_0 = a_0, ..., a_l$ with $0 \leq l < \lfloor \frac{|S|}{3} \rfloor$, $\texttt{code}^{-1}(a_0)$ is a set of ordered triples with the same middle component according to prerequisite ($\star$).

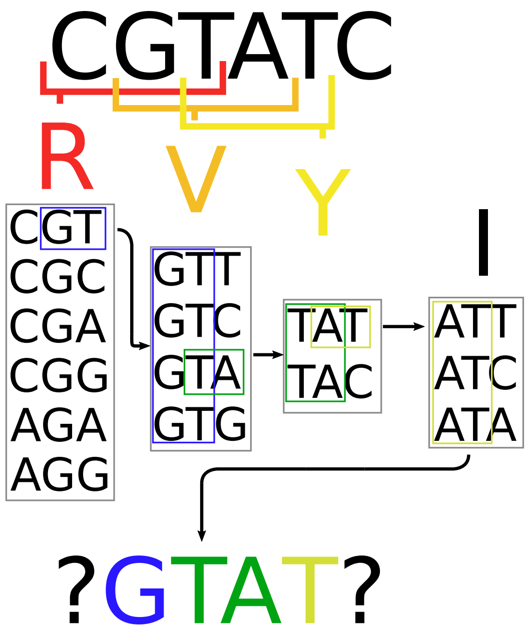

Figure 4: Translation and inversion.

Following observation 5.0.1, the sets created by applying $\texttt{code}^{-1}$ to the letters of w_0, w_1 and w_2 must contain at least one triplet, where the bases match in the described positions (see Figure 4).

Repeating this chained matching and checking reveals all interior bases of S. Since the first base in the first frame and the last base of the last frame are not necessarily unique and cannot be checked by the other frames, they are considered ambiguous. Therefore the sequence S can be reconstructed with just the amino-acid-like encoded frames except for the first and last base. □

Remark 5.1.1. *The standard codon table cannot be constructed with* `code`*, because prerequisite* ($\star$) *is not satisfied ("S" has two middle bases). To fix that, one must first split the amino acid "S" into two letters ("AGT" and "AGC") and second give the stop codon "TGA" an additional letter as well. The default codon table used in kASA implements the additional stop codon but does not introduce a new letter to split "S" to be compatible with already converted amino acid sequences. Benchmark results for both the standard codon table and one with a split "S" did not differ noticeably so an approximation of* `code` *(by the standard codon table) is sufficient.*

Remark 5.1.2. *One can reconstruct the first and last base of* S *if we additionally restrict the alphabet* $\mathcal{A}$ *further: The first base of every triplet must be unique to the assigned letter* $a \in \mathcal{A}$*. Secondly, six instead of three frames are*

used (so the reverse complement is translated as well). A possible function **code** *using a 16-letter alphabet maps each combination of the first two bases in a triplet to a unique letter, so*

$$\texttt{code}_{16}((b_0, b_1, b_2)) = code_{16}((c_0, c_1, c_2)) \Rightarrow b_0 = c_0 \text{ and } b_1 = c_1,$$

$$(b_0, b_1, b_2),\ (c_0, c_1, c_2) \in \mathcal{B} \times \mathcal{B} \times \mathcal{B}.$$

Remark 5.1.3. *During the proof, we made the assumption that the ordering of the frames are as* **translate** *created them. However, should this ordering be disturbed we can try to use the constructed* **translate**$^{-1}$ *anyway because the reconstruction will fail if the ordering is not correct. This is because no other combination would generate S in full length in the reconstruction process.*

Remark 5.1.4. *This proof offers another insight: Even though the frames together contain almost all information of the DNA sequence, the k-mers themselves do not. This means that one amino acid-like encoded k-mer may very well match erroneously and thus decrease precision. This can be seen when using a different coding alphabet like the 16 letter alphabet described in 5.1.2. It implies that every third letter of the sequence is ignored for each frame which leads to higher robustness but also reduced precision. We therefore assume this to be case of: "The sum is bigger than its parts" and if the context which k-mer belongs to which frame is destroyed during lexicographical sorting, we might lose too much information and thus suffer a decrease in precision. We suspect that the standard conversion alphabet performs well because there might be an intrinsic balance between information conservation and robustness for each k-mer in it. To draw final conclusions, further study is needed.*

An implementation of the constructive proof can be found in `https://github.com/SilvioWeging/kASA/tree/master/scripts/reconstructDNA.py`.

5.1 Experiments with different alphabets

We did some experiments trying to determine to which degree a different conversion table influences the accuracy and robustness. First, we sampled a few bacterial genomes and randomly created alphabets that comply to the definition of `code` with lengths from 8 to 27 (the highest number of letters allowed for an alphabet used within kASA) and three variants each. We also included the 16 letter alphabet (always on the 16 tick) mentioned in remark 5.1.2 as well as the standard codon table (red dot).

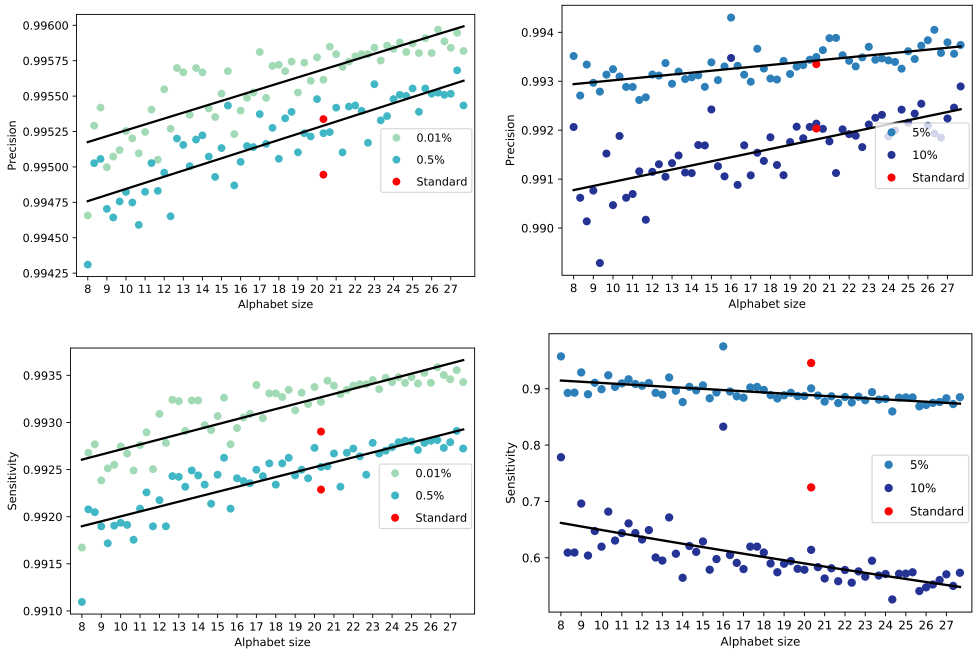

We see that using more letters results in a higher precision and sensitivity while the number of mutations are low. After a certain percentage of mutations, the sensitivity is affected lowering the robustness. The 16 letter alphabet as well as the standard codon table seem to perform better with more mutations but worse with less to almost no mutation. To further test this we took one of the 27 letter alphabets as well as the 16 and standard codon table and tested them with the robustness benchmark.

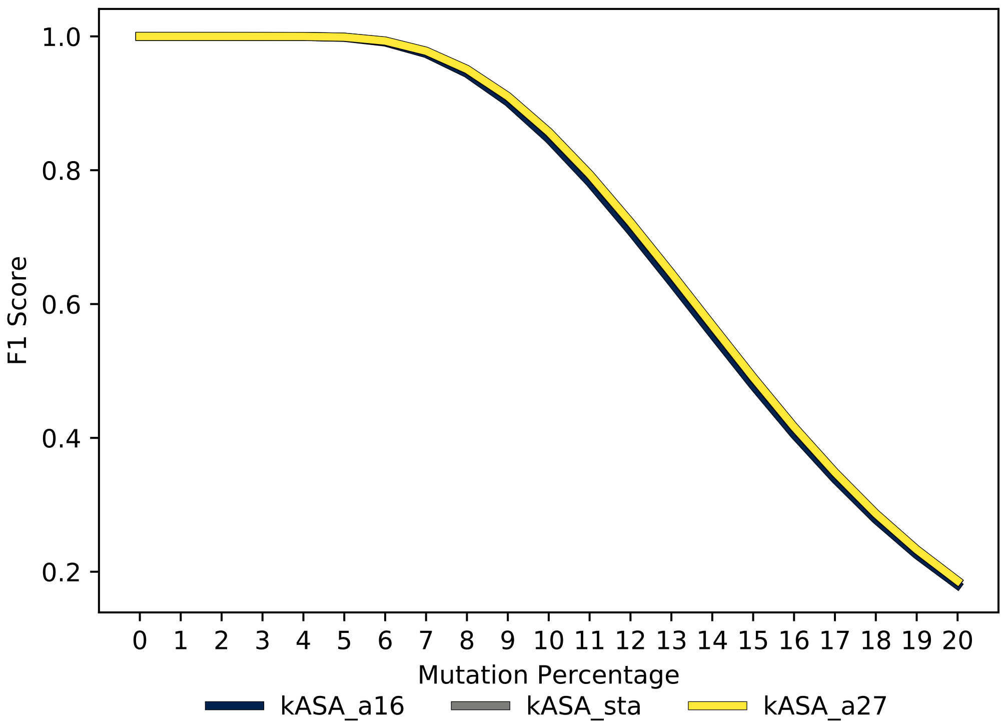

We see that the effect on the robustness is negligible if eukaryotic data is added. We thus keep the standard codon table a default in kASA for translating from DNA to our amino acid-like encoding.

References

[1] Alexa B. R. McIntyre, Rachid Ounit, Ebrahim Afshinnekoo, Robert J. Prill, Elizabeth Hénaff, Noah Alexander, Samuel S. Minot, David Danko, Jonathan Foox, Sofia Ahsanuddin, Scott Tighe, Nur A. Hasan, Poorani Subramanian, Kelly Moffat, Shawn Levy, Stefano Lonardi, Nick Greenfield, Rita R. Colwell, Gail L. Rosen, and Christopher E. Mason. Comprehensive benchmarking and ensemble approaches for metagenomic classifiers. *Genome Biology*, 18(1):182, Sep 2017.

[2] Stinus Lindgreen, Karen L. Adair, and Paul P. Gardner. An evaluation of the accuracy and speed of metagenome analysis tools. *Scientific Reports*, 6:19233 EP –, Jan 2016. Article.

[3] Daehwan Kim, Li Song, Florian P. Breitwieser, and Steven L. Salzberg. Centrifuge: rapid and sensitive classification of metagenomic sequences. *Genome Research*, 26(12):1721–1729, 2016.

[4] Peter Menzel, Kim Lee Ng, and Anders Krogh. Fast and sensitive taxonomic classification for metagenomics with kaiju. *Nature Communications*, 7:11257 EP –, Apr 2016. Article.

[5] M. Burrows and D. J. Wheeler. A block-sorting lossless data compression algorithm. Technical report, 1994.

[6] P. Ferragina and G. Manzini. Opportunistic data structures with applications. In *Proceedings 41st Annual Symposium on Foundations of Computer Science*, pages 390–398, Nov 2000.
